# An Integrated Analysis of Radial Spoke Head and Outer Dynein Arm Protein Defects and Ciliogenesis Abnormality in Nasal Polyps

**DOI:** 10.3389/fgene.2019.01083

**Published:** 2019-11-13

**Authors:** Xiao-xue Zi, Wei-jie Guan, Yang Peng, Kai Sen Tan, Jing Liu, Ting-ting He, Yew-kwang Ong, Mark Thong, Li Shi, De-yun Wang

**Affiliations:** ^1^Department of Otolaryngology-Head and Neck Surgery, Shandong Provincial ENT Hospital Affiliated to Shandong University, Jinan, China; ^2^Department of Otolaryngology, Yong Loo Lin School of Medicine, National University of Singapore, Singapore, Singapore; ^3^State Key Laboratory of Respiratory Disease, National Clinical Research Center for Respiratory Disease, Guangzhou Institute of Respiratory Health, First Affiliated Hospital of Guangzhou Medical University, Guangzhou Medical University, Guangzhou, Guangdong, China; ^4^Department of Otolaryngology-Head and Neck Surgery, National University Hospital System (NUHS), Singapore, Singapore

**Keywords:** abnormal ciliary ultrastructure, inflammation, inferior turbinate, nasal polyps, mucociliary clearance, up-regulated ciliogenesis

## Abstract

**Background:** Nasal polyp (NP) is a chronic upper airway inflammatory disease that is frequently triggered by defective host-defense. However, the mechanisms underlying the impaired barrier function such as cilia-mediated mucociliary clearance remain poorly understood.

**Objective:** To assess ciliary ultrastructural and ciliogenesis marker expression and the phenotypes of ciliated cells in NP.

**Methods:** NP biopsy samples were obtained from 97 NP patients and inferior turbinate from 32 healthy controls. Immunofluorescence staining, quantitative polymerase chain reaction, and single-cell cytospin staining were performed. We classified the patterns of radial spoke head protein (*RSPH*) 1, 4A (*RSPH4A*), 9 (*RSPH9*), and dynein axonemal heavy chain 5 (*DNAH5*) localization. A semi-quantitative scoring system was developed to assess their expression patterns and associations with ciliogenesis markers [centrosomal protein 110 (*CP110*) and forkhead box j1 (*FOXJ1*)].

**Results:** Median scores of *RSPH1*, *RSPH4A*, *RSPH9,* and *DNAH5* were significantly higher in NP than in healthy controls, particularly in eosinophilic NPs. Expression pattern scores of *RSPH1*, *RSPH4A*, *RSPH9,* and *DNAH5* correlated positively with each other in both groups. In primary-cell specimens, abnormal expression patterns were significantly more common in NP. The total fluorescence intensity of *CP110* and *FOXJ1* was significantly higher in NPs and correlated positively with expression pattern scores of *RSPH1*, *RSPH4A*, *RSPH9,* and *DNAH5*. A trend towards lengthened cilia was observed in NP.

**Conclusion:** In the chronic airway inflammatory milieu, the up-regulated ciliogenesis correlates with the abnormal expression of ciliary ultrastructural markers (i.e., *DNAH5*) in NP (particularly eosinophilic NP).

## Introduction

Nasal polyp (NP) is a common chronic upper airway inflammatory disorder that frequently co-exists with lower airway inflammatory diseases such as asthma (Fokkens et al., 2012). In Asian population, NP is mostly characterized by prominent epithelial remodeling and mixed inflammatory phenotypes (Hao et al., 2006). Physiologically, the respiratory cilia maintain proper clearance of pathogens and allergens (Li et al., 2012). We have previously demonstrated the poorly proliferated basal cells and up-regulation of ciliogenesis markers [centrosomal protein 110 (*CP110*), and fork-head box J1 (*FOXJ1*)] in the aberrantly remodeled epithelium of NP (Li et al., 2014; Zhao et al., 2017). Furthermore, abnormal ciliary morphology (i.e., overly dense and lengthened cilia), abnormal expression of dynein axonemal heavy chain 5 (*DNAH5*, the marker crucial to microtubule sliding), and the significantly reduced ciliary beat frequency (CBF) have also been observed in NP (Lai et al., 2011;Li et al., 2014; Qiu et al., 2018). Therefore, abnormal ciliogenesis and/or ultrastructure might be critical drivers of impaired mucociliary clearance (MCC), contributing to the chronic inflammation in NPs.

Physiologically, ciliary motility is mainly regulated by the outer dynein arms and radial spoke (RS) complexes (Smith and Yang, 2004; Frommer et al., 2015). Abnormal expression of *DNAH5* (which has previously been linked to genetic defects) has been implicated in chronic lower airway inflammatory diseases such as primary ciliary dyskinesia (PCD), Kartagener syndrome, bronchiectasis, and chronic obstructive pulmonary disease (Hornef et al., 2006; Failly et al., 2009; Leigh et al., 2009; Lee et al., 2014; Chen et al., 2018; Qiu et al., 2018). RSs are important ultrastructural proteins that are aligned between the central and peripheral microtubules (Shinohara et al., 2015). Mutations of RS head protein 1 (*RSPH1*), 4A (*RSPH4A*), and 9 (*RSPH9*) could have significantly affected the interactions between RS head and central microtubules (Frommer et al., 2015). Intriguingly, RS defects have been identified among 19% of patients with ciliary ultrastructural abnormalities (Plesec et al., 2008), and reportedly account for ∼6% of patients with PCD (Ibanez-Tallon et al., 2003).

To ensure proper ciliary motility, ciliogenesis is crucial to the formation of axonemes where ciliary ultrastructural proteins are assembled. *CP110* reportedly regulated centrosome duplication and centriole conversion to basal bodies (Chen et al., 2002;Yadav et al., 2016). Inflammation-mediated up-regulation of *CP110* contributed to defective cilia assembly and decreased motility in CRS (Lai et al., 2011;Li et al., 2014). Additionally, increased FOXJ1-positive cell count correlated with longer and denser cilia in NP (Li et al., 2014). Collectively, abnormal expression of *CP110* and *FOXJ1* may have resulted in disrupted cilia assembly in NP.

Currently, most reports focused on isolated ciliogenesis or ciliary ultrastructural marker. The association between ciliogenesis or ciliary ultrastructural marker expression remains understudied. Furthermore, abnormal expression of some ultrastructural markers (i.e. *DNAH5*) was reportedly present in congenital diseases such as PCD. Because secondary ciliary dyskinesia (SCD) is common among various chronic inflammatory diseases (Al-Rawi et al., 1998), we hypothesized that the inflammatory milieu might be the critical driver of the defective ciliogenesis and abnormality of ultrastructural markers in NP. Building on our previous research, we sought to systematically investigate the expression patterns of four ciliary ultrastructural markers (*RSPH1*, *RSPH4A*, *RSPH9,* and *DNAH5*) and two ciliogenesis markers (*CP110* and *FOXJ1*) for their manifestation of ciliary impairment in NPs. Our findings might help elucidate the roles of ciliogenesis and ciliary ultrastructural markers in NP pathogenesis, and whether chronic airway inflammation is responsible for the abnormal expression of ciliary ultrastructural markers.

## Methods

### Study Population

Surgical samples from 97 patients with NP and inferior turbinate (IT) mucosa from 32 control subjects were obtained from The Second Affiliated Hospital of Shandong University (China) and National University Hospital of Singapore (Singapore). Patients were diagnosed as having chronic rhinosinusitis with NP (grade 2 or 3) according to European Position Paper on Rhinosinusitis and Nasal Polyps 2012 criteria and NP was histologically confirmed post-operatively. (Fokkens et al., 2012). Patients with upper respiratory tract infections within four weeks and who were highly suspicious of having PCD were excluded. Atopy was evaluated with skin prick testing, and asthma was diagnosed based on physician’s diagnosis. Control subjects were scheduled for septum plastic surgery and remaining free of sinus symptoms. Primary single-cell specimens for cytospin were obtained from NP (n = 20) and healthy controls (n = 11). Due to the limited sizes of the tissue, not all specimens were used for each analysis.

Our study was carried out in accordance with The Declaration of Helsinki. Ethics approval was obtained from the institutional review boards of the two participating hospitals. All participants signed written informed consent.

### Cytospin Preparation

Single-cell suspensions were obtained from fresh nasal specimens by using enzymatic digestion and mechanical dissociation. Sample was treated in 10 mg/ml of Dispase II (Sigma-Aldrich Inc., USA) followed by gentle shaking for 4°C overnight incubation. We treated samples with 1× trypsin/ethylene diaminetetraacetic acid at 37°C for 15 min. The dissociated cells were fixed in 4% formaldehyde at room temperature for 10 min, and washed twice with 1× Dulbecco’s phosphate-buffered saline. Cytospin (1-2×10^4^ cells/slide) was prepared at 500 rpm for 5 min by using Shandon Cytospin 3 Cytocentrifuge (Thermo Fisher Scientific, Waltham, MA).

### Immunohistochemistry (IHC) and Immunofluorescence (IF) Assays

Paraffin tissue sections and cytospin samples were used to perform the IHC and IF assay. See **Online Supplementary Text**for details.

### Evaluation of IHC and Immunofluorescence Assays

#### Eosinophil and Neutrophil Count

All cases were assessed in a blinded fashion by independent researchers. Eosinophil infiltration was evaluated based on haemotoxylin-eosin (H&E) staining, while neutrophil infiltration by IHC staining. Eosinophils and neutrophils were enumerated at five high-power fields (HPF, 400× magnification) with the infiltration of inflammatory cell. Eosinophilia or neutrophilia denoted eosinophils or neutrophils exceeding 10% of the total leucocyte count (Gao et al., 2016).

#### Measurement of Cilia Length

Primary single cells from 20 patients with NP and 11 healthy controls were subject to cytospin slide preparations. Cilia length assessment was based on alpha-tubulin staining. We randomly selected five areas of images at 400× magnification. Cilia length was measured with ImageJ software and 20 measurements for each area were recorded. Scores of individual measurements were averaged before analysis.

#### RSPH1, RSPH4A, RSPH9, and DNAH5 Expression Patterns

Merged images were used for analyzing expression patterns of ciliary ultrastructural and ciliogenesis markers. Based on previous publications, immunofluorescence imaging of *RSPH1*, *RSPH4A*, *RSPH9,* and *DNAH5* shared three patterns (Shoemark et al., 2017; Qiu et al., 2018): (i) Pattern A, markers located throughout the entire axoneme; (ii) Pattern B, markers partly missing at distal axoneme. (iii) Pattern C, markers completely missing throughout the axoneme. To determine the magnitude of abnormality of ciliary ultrastructural markers, we developed a semi-quantitative scoring system for which 0 denoted pattern A > 70%, 1 denoted patterns A + B > 70%, and 2 denoted pattern C ≥ 30% to assess co-localization. Three to five areas of merged images at 400× magnifications were selected randomly, and scores of individual measurements were averaged (Shoemark et al., 2017; Qiu et al., 2018). In cytospin slides, 10 single cilia cells were randomly selected from each sample and the expression patterns of single cells were graded by using oil-immersion lens at 1,000× magnifications.

#### Evaluation of CP110 and FOXJ1 Expression

The total fluorescence intensity (TFI, presented in arbitrary units) measurements were performed to evaluate *CP110* and *FOXJ1* expression. The IF images on tissue sections were captured with 40× objective lens of fluorescence microscope (Olympus IX51; Olympus, Nagano, Japan). The positively stained area and mean fluorescence intensity (MFI) of each marker were recorded using ImageJ software. The TFI was calculated as the product of the positively stained area and the MFI (Li et al., 2014).

### RNA Extraction and Quantitative Real-Time Polymerase Chain Reaction (qRT-PCR)

Total RNA was extracted from NP and IT in RNAlater (Ambion, Austin, TX, USA) with mirVana^™^ isolation kit. Expression of markers was evaluated by performing qRT-PCR (9StepOnePlusTM System, Applied Biosystems Inc., USA). Relative gene expression was calculated using the 2^-ΔΔCt^ algorithm with glyceraldehyde-3-phosphate dehydrogenase as the reference. Details are presented in **Online Supplementary Text**.

### Transmission Electron Microscopy (TEM)

Tissue samples from patients with NP and controls were prepared according to the standardized protocol of performing the TEM. The cilia ultrastructure was evaluated with the JEOL instrument (Model: JEM 1010, Jeol Co. Ltd., Japan). Further details are presented in the **Online Supplementary Text**.

### Statistical Analysis

Analyses were conducted using SPSS 22.0 (SPSS Inc., USA) and GraphPad Prism 6.0 (GraphPad Inc., USA). Differences in categorical variables (e.g., epithelial hyperplasia and eosinophilia/neutrophilia), *RSPH1*, *RSPH4A*, *RSPH9,* and *DNAH5* expression patterns of single-cell cytospin preparations between two groups were compared with chi-square test or Fisher’s exact test as appropriate. The between-group difference in ciliary marker expression was analyzed with Mann-Whitney U test. The correlation analysis was performed by Spearman’s correlation model and bootstrapping analysis. Linear mixed models were applied to compare the cilia length from primary cytospin preparations. P ≤ 0.05 was considered significant for all analyses.

## Results

### Demographic and Clinical Characteristics

The demographic and clinical characteristics are summarized in **Table 1**. Despite a trend towards the greater age in patients with NP, the percentage of males and participants with atopy and asthma was comparable. Compared with IT, eosinophilic and neutrophilic infiltration was markedly more prevalent in patients with NP (6.3% vs. 45.4%; 9.4% vs. 59.8%, both *P* < 0.001). Epithelial hyperplasia was more common among patients with NP (84.5% vs. 9.4%, *P* < 0.001).

**Table 1:** Demographic and clinical characteristics of study participants.

Parameters	Controls	CRSwNP	*P* value
**Nasal tissue (No.)**	32	97	–
Paraffin specimens for staining (No.)	32	97	–
RNA for qRT-PCR (No.)	28	62	–
Cytospin preparations for staining (No.)	11	20	–
**Age, yr [mean ± SD]**	33.8 ± 14.3	40.2 ± 16.5	0.057
**Male [N (%)]**	22 (68.8)	67 (69.1)	0.973
**Atopy [N (%)]**	7 (21.9)	20 (20.6)	0.880
**Asthma [N (%)]**	2 (6.3)	18 (18.6)	0.157
**Epithelial hyperplasia** **^†^**	**3 (9.4)**	**82 (84.5)**	**<0.001**
**Inflammatory cells [N (%)]** **^‡^**			
Eosinophilia (No., %)	**2 (6.3)**	**44 (45.4)**	**<0.001**
Neutrophilia (No., %)	**3 (9.4)**	**58 (59.8)**	**<0.001**

CRSwNP, Chronic rhinosinusitis with nasal polyps; NA, not applicable; SD, standard deviation; yr, year; RNA, ribonucleic acid; qRT-PCR, quantitative reverse transcriptase polymerase chain reaction

Differences in categorical variables between the two groups were compared using chi-square test or Fisher’s exact test. The difference of age was compared with independent t-test.

^†^Epithelium with more than 4 layers was defined as epithelial hyperplasia.

^‡^The percentage of eosinophils and neutrophil exceeding 10% was categorized as eosinophilia or neutrophilia.

P < 0.05 was considered statistically significant unless otherwise stated. Data in bold indicated the statistical analysis with significance.

### Aberrant Expression of Ciliary Ultrastructural Markers in NPs

**Figure 1:**
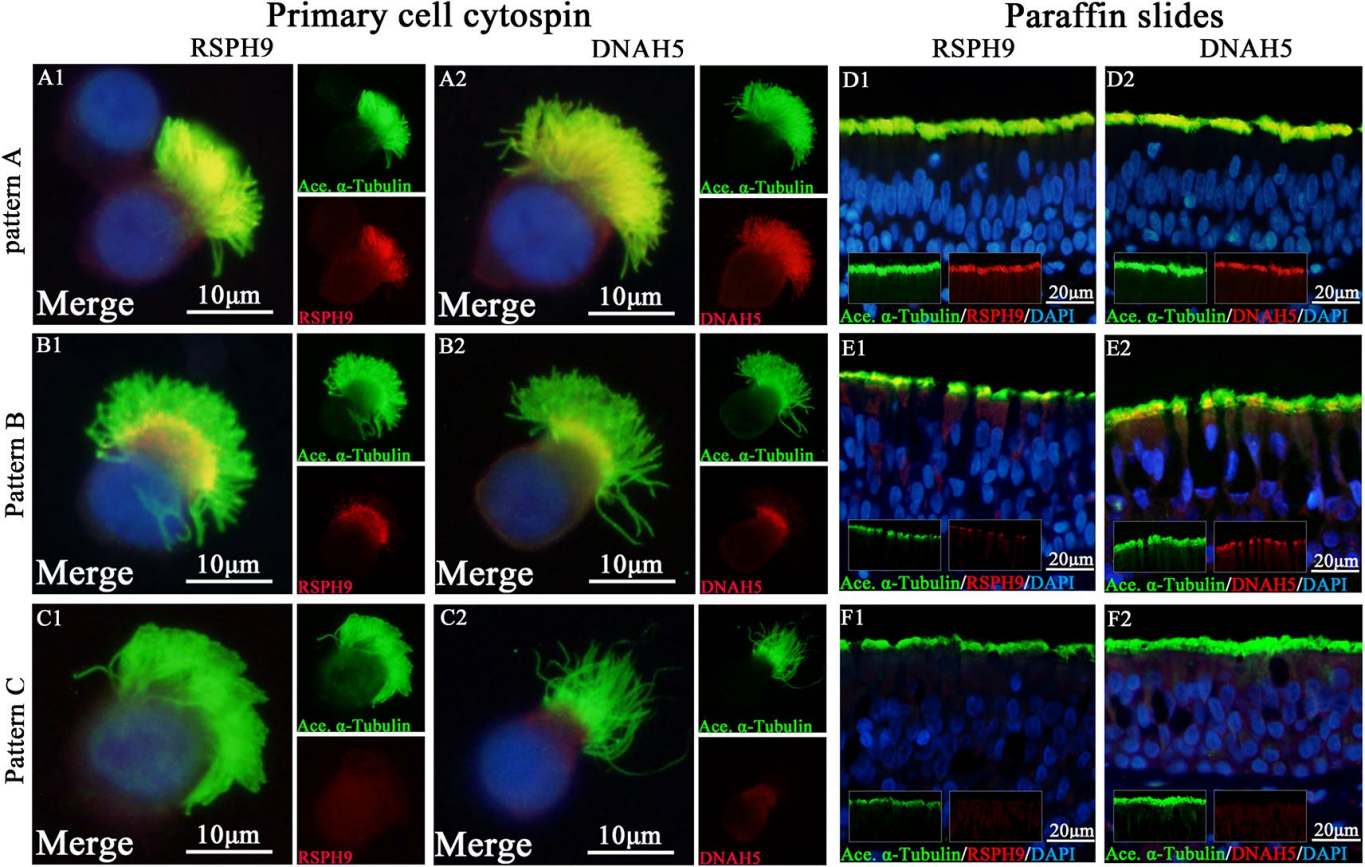
Expression patterns of *RSPH9* and *DNAH5* in primary single-cell cytospin slides and paraffin slides. *RSPH1*, *RSPH4A*, *RSPH9,* and *DNAH5* shared three patterns based on our previous report, which were defined as Pattern **(A)**, markers located throughout the entire axoneme (A1-A2, D1-D2); Pattern **(B)**, markers partly missing at the distal parts of axoneme (B1-B2, E1-E2). Pattern **(C)**, markers completely missing throughout the entire axoneme (C1-C2, F1-F2). The expression patterns of RSPH1 and RSPH4A have been demonstrated in **Figure S1**. *RSPH9* and *DNAH5, Red*; *alpha-tubulin*, Green; DAPI, Blue; Co-localization of *RSPH1*, *RSPH4A*, *RSPH9,* and *DNAH5* with alpha-tubulin, Yellow. RSPH, Radial spoke head protein; DNAH5, Dynein arm heavy chain 5; DAPI, 4’, 6-diamidino-2-phenylindole.

We initially determined the co-localization of *RSPH1*, *RSPH4A*, *RSPH9*, *DNAH5,* and acetylated alpha-tubulin. Consistent with our previous reports, three patterns (Pattern A-C) of *RSPH1*, *RSPH4A*, *RSPH9,* and *DNAH5* were observed in both paraffin sections and primary single-cell cytospin slides (**Figure 1**).

**Figure 2:**
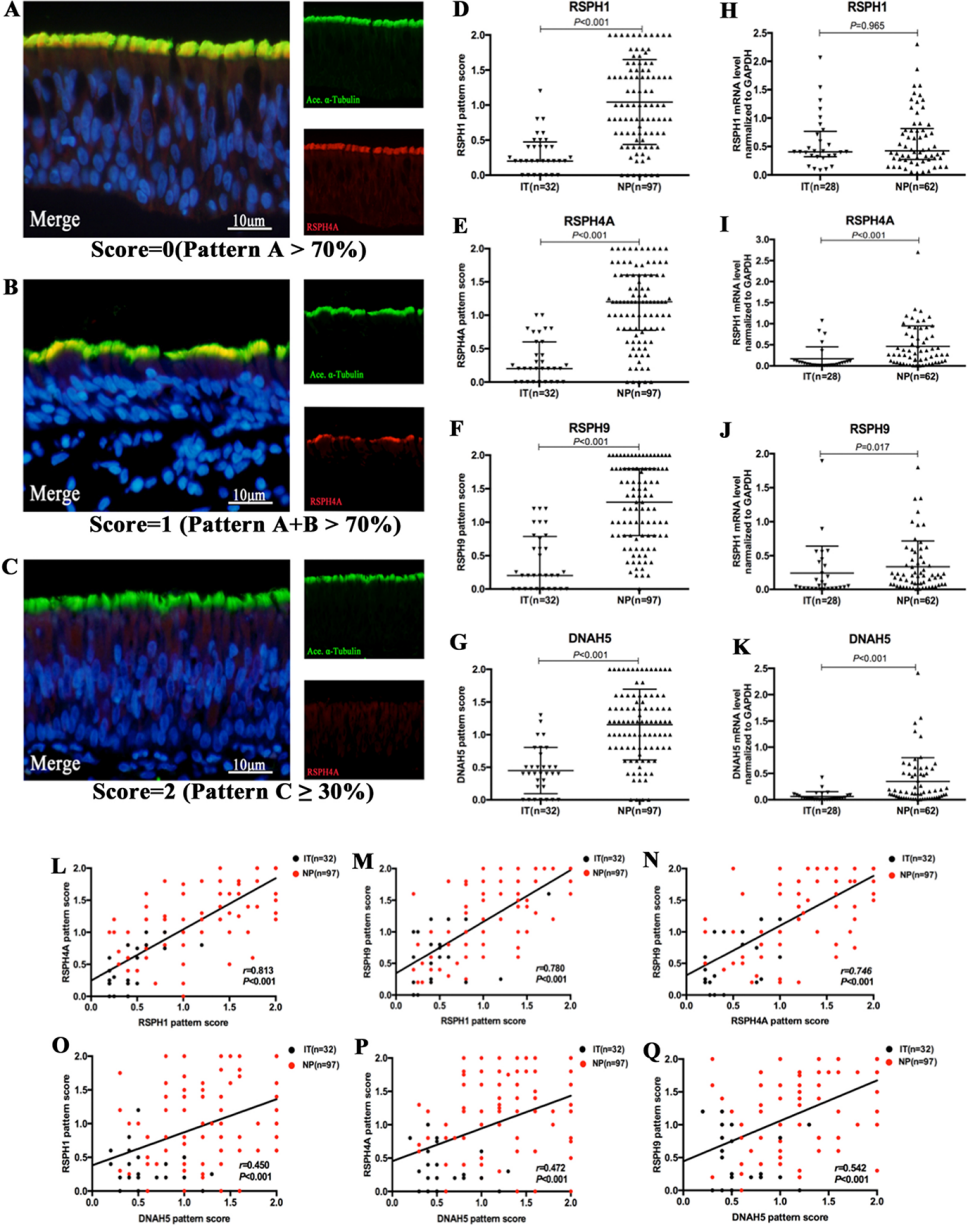
The semi-quantitative grading system for paraffin specimens, the abnormal expressions of ciliary ultrastructural markers and their association in nasal epithelial specimens. **(A**–**C)** A semi-quantitative scoring system for which 0 = pattern A > 70% **(A)**; 1 = patterns A + B > 70% **(B)**; and 2 = pattern C ≥ 30%**(C)** to assess the co-localizations in paraffin. **(D**–**K)** The pattern scores **(D**–**G)** and the mRNA expressions **(H**–**K)** of *RSPH1*, *RSPH4A*, *RSPH9,* and *DNAH5*. **(L**–**Q)** Correlation between the pattern scores of *RSPH1*, *RSPH4A*, *RSPH9,* and *DNAH5. RSPH1*, *RSPH4A*, *RSPH9,* and *DNAH5, Red*; *alpha-tubulin*, Green; DAPI, Blue; Co-localization of *RSPH1*, *RSPH4A*, *RSPH9,* and *DNAH5* with alpha-tubulin, Yellow. RSPH, Radial spoke head protein; DNAH5, Dynein arm heavy chain 5; DAPI, 4’,6-diamidino-2-phenylindole.

Based on our semi-quantitative scoring system, the median (the 1^st^ and 3^rd^ quartile) scores of *RSPH1*, *RSPH4A*, *RSPH9,* and *DNAH5* were 0.2 (0.2, 0.5), 0.2 (0, 0.6), 0.2 (0, 0.8), and 0.4 (0.2, 0.7) in IT for paraffin sections, respectively. Conversely, the median (the 1^st^ and 3^rd^ quartile) scores of *RSPH1*, *RSPH4A*, *RSPH9,* and *DNAH5* were significantly higher [1.0 (0.6, 1.5), 1.2 (0.8, 1.6), 1.3 (0.8, 1.8), and 1.2 (0.8, 1.6)] in patients with NPs (all *P* < 0.001). In addition, mRNA expression levels of *RSPH4A* (*P* < 0.001), *RSPH9* (*P* = 0.017), and *DNAH5* (*P* < 0.001) were significantly higher in NP than in IT, although mRNA expression of *RSPH1* was comparable (*P* = 0.965) (**Figure 2**).

In single-cell cytospin slides, the percentage of pattern A-C was 50.0%, 22.0%, and 28.0% for *RSPH1*, 58.0%, 12.0%, and 30.0% for *RSPH4A*, 48.0%, 18.0%, and 34.0% for *RSPH9*, and 51.5%, 13.5%, and 35.0% for *DNAH5* in patients with NP, respectively. Conversely, the percentage of patterns A-C among control subjects was 72.0%, 18.0%, and 10.0% for *RSPH1*, 88.0%, 6.0%, and 6.0% for *RSPH4A*, 72.0%, 12.0%, and 16.0% for *RSPH9*, and 74.0%, 14.0%, and 12.0% for *DNAH5*, respectively (all *P* < 0.05). (**Table 2**)

**Table 2:** The expression patterns of ciliary ultrastructural markers in primary cell specimens.

Immunofluorescence staining patterns	Controls	CRSwNP	*P* value
**Ciliary markers staining (No.)**	5	5	–
**Single ciliated cells evaluated (No.) ** **^#^**	50	50	–
**RSPH1 staining patterns [No. (%)]**	–	–	**0.040**
Pattern A	36 (72.0)	25 (50.0)	–
Pattern B	9 (18.0)	11 (22.0)	–
Pattern C	5 (10.0)	14 (28.0)	–
**RSPH4A staining patterns [No. (%)]**	–	–	**0.002**
Pattern A	44 (88.0)	29 (58.0)	–
Pattern B	3 (6.0)	6 (12.0)	–
Pattern C	3 (6.0)	15 (30.0)	–
**RSPH9 staining patterns [No. (%)]**	–	–	**0.044**
Pattern A	36 (72.0)	24 (48.0)	–
Pattern B	6 (12.0)	9 (18.0)	–
Pattern C	8 (16.0)	17 (34.0)	–
**Ciliary markers staining (No.) ** **^§^**	5	20	–
**Single ciliated cells evaluated (No.) ** **^#^**	50	200	–
**DNAH5 staining patterns [No. (%)]**	–	–	**0.005**
Pattern A	37 (74.0)	103(51.5)	–
Pattern B	7 (14.0)	27(13.5)	–
Pattern C	6 (12.0)	70(35.0)	–

The number of patient’s samples for immunostaining of different ultrastructural markers was variable because of the limited cell count for performing assays. Priority was assigned to DNAH5 staining.

^#^Primary cells were obtained from 5 patients, and 10 single ciliated cells were randomly selected for assessment from each sample.

^§^Primary cells were obtained from 5 healthy controls and 20 patients with nasal polyps, 10 single ciliated cells were randomly selected for assessment from each sample.

P value in bold indicated the statistical analysis with significance.

RSPH, Radial spoke head protein; DNAH5, Dynein axonemal heavy chain 5.

### Ciliogenesis Marker Expression

**Figure 3:**
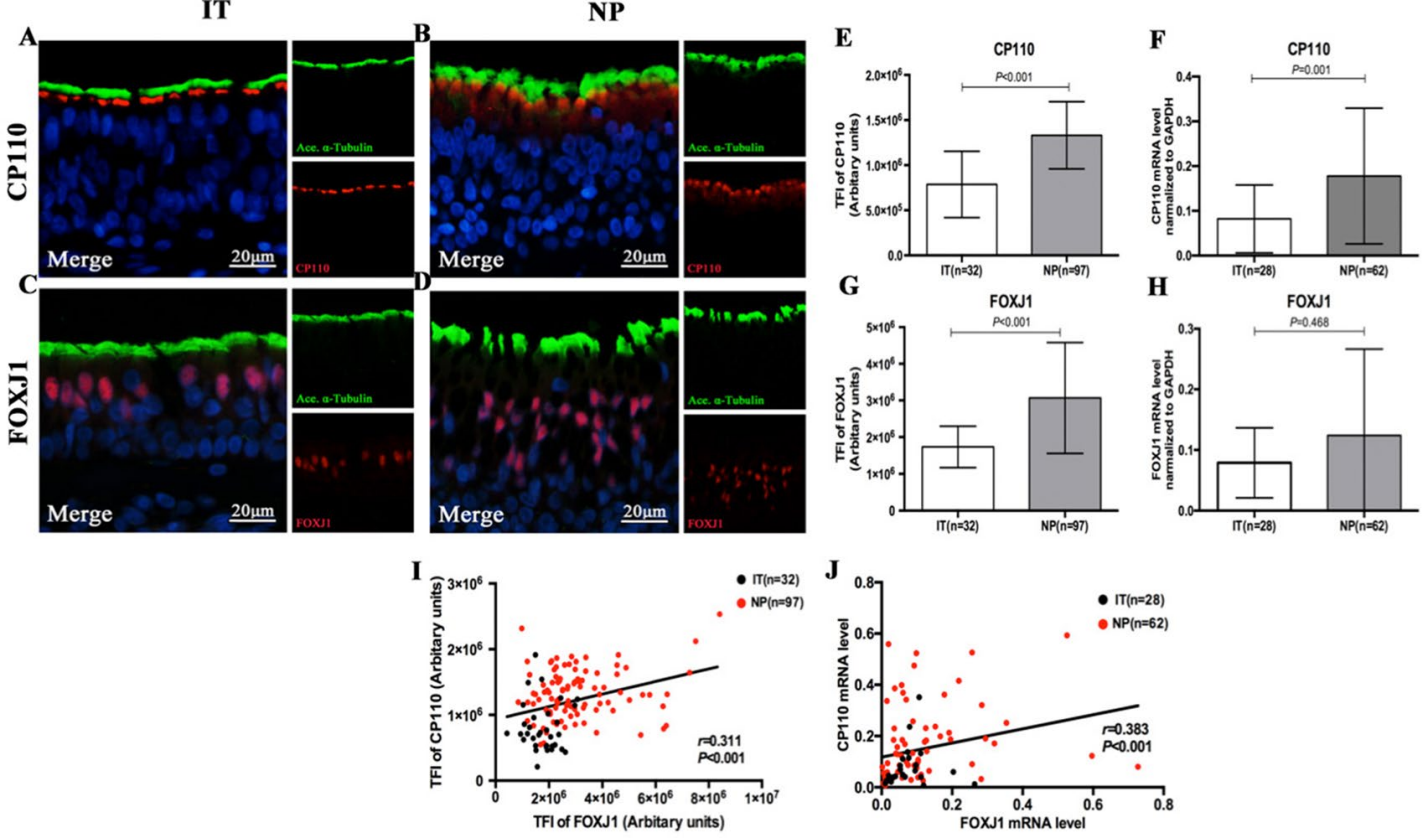
Comparison of the expression levels of *CP110* and *FOXJ1* in patients with nasal polyps and healthy controls and their correlation. Shown in the figure are the expression levels of CP110 **(A–B, E–F)** and FOXJ1 **(C–D, G–H)** in patients with nasal polyps and healthy controls. CP110 staining showed a localized and thin pattern in control subjects, while a diffuse and thick pattern was seen in NP. FOXJ1 was stained within the nucleus of ciliated and non-ciliated epithelial cells. The correlation between FOXJ1 and CP110 expression levels is demonstrated in **Figure 3I–J**. *CP110* and *FOXJ, Red*; *alpha-tubulin*, Green; DAPI, Blue. *CP110*, Centrosomal protein 110; *FOXJ*, Fork-head box protein J1, DAPI, 4’,6-diamidino-2-phenylindole.

The IF staining patterns and the intensity of *CP110* in patients with NP differed considerably from those in IT tissues in control subjects. *CP110* staining showed a localized and thin pattern in control subjects, while a diffuse and thick pattern was seen in NP. The median (the 1^st^ and 3^rd^ quartile) of TFI for *CP110* was significantly higher in NP than in IT [1.3 (1.1, 1.6) vs. 0.7 (0.5, 0.9) ×10^6^ arbitrary units, *P* < 0.01]. Consistently, *CP110* mRNA levels were considerably higher in NP biopsy tissues than in IT (*P* = 0.001) (**Figure 3**).

*FOXJ1* was stained within the nucleus of ciliated and non-ciliated epithelial cells. Compared with control subjects, the TFI for *FOXJ1* staining was markedly greater in NP than in IT [2.7 (2.1, 3.7) vs. 1.7 (1.3, 2.1) ×10^6^ arbitrary units, *P* < 0.01]. However, mRNA expression of *FOXJ1* was non-significantly greater in NP (*P* = 0.468) (**Figure 3**).

### Association Between Ciliary Ultrastructural and Ciliogenesis Markers

The IF expression pattern scores of *RSPH1*, *RSPH4A*, *RSPH9,* and *DNAH5* correlated positively with each other in biopsy tissues of both groups (all *P* < 0.001) (**Figure 2**). Similarly, the mRNA expression levels showed similar correlation to the pattern scores (all *P* < 0.01) (**Figure S2**). The same trends were found in the NP-only groups (all *P* < 0.05) (Figure S3). The correlations were further confirmed based on the chi-square analysis that showed no significant difference between the normal/abnormal distribution among different ciliary markers. Similar findings were also observed using the bootstrapping analysis (**Tables S3**and **S4**).

**Figure 4:**
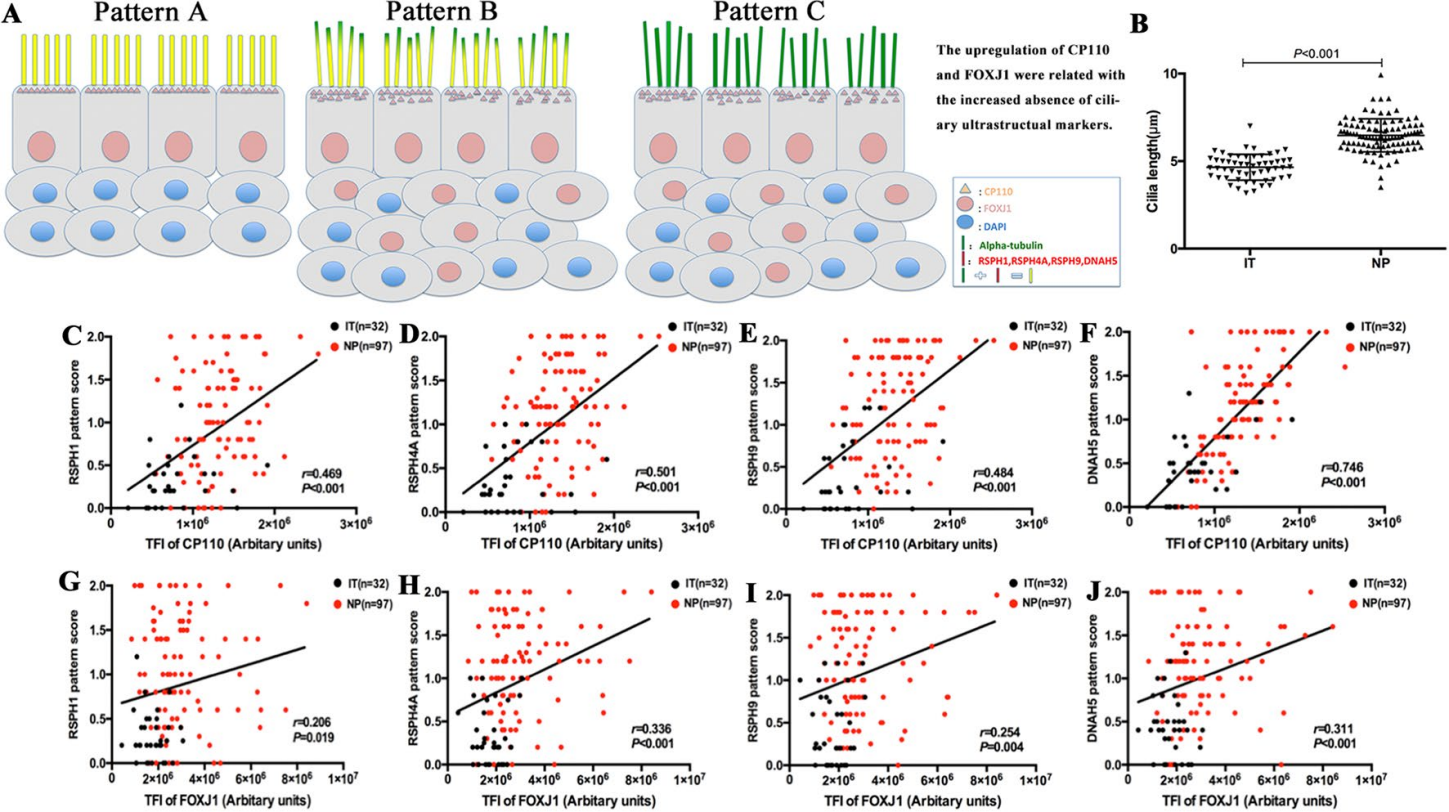
A schematic diagram demonstrating the different patterns of ciliary disorders, the difference of length between NPs and ITs, and the correlation between ciliary ultrastructural markers and ciliogenesis markers. Shown in the figure is a schematic diagram demonstrating the different expression patterns of ciliary disorders and the relevant changes in the expression of ciliogenesis markers and ultrastructural markers **(A)**. Pattern A is the normal baseline. Up-regulation of *CP110* and *FOXJ1* are associated with the greater cilia length and increased absence of ciliary ultrastructural markers in Pattern B and Pattern C. Linear mixed models were employed to compare between healthy controls (n = 13) and patients with NPs (n = 20) **(B)**. The correlation between the expression of *CP110* and ciliary ultrastructural markers **(C**–**F)**. Data from IT tissues and NP tissues appeared in black and red dots, respectively. The correlation between the expression of *FOXJ1* and ciliary ultrastructural markers **(G**–**J)**. Data from IT tissues and NP tissues appeared in black and red dots, respectively. *RSPH*, Radial spoke head protein; *DNAH5*, Dynein arm heavy chain 5; *CP110*, Centrosomal protein 110; *FOXJ*, Fork-head box protein J1, DAPI, 4’,6-diamidino-2-phenylindole.

Both protein expression levels (assessed with TFI) and mRNA expression of *CP110* and *FOXJ1* were correlated positively in biopsy samples (*r* = 0.311, *P* < 0.001; *r* = 0.383, *P* < 0.001, **Figures 3** and **S2**). We further investigated the associations of the expression levels between ciliary ultrastructural markers and ciliogenesis markers. The TFI of *CP110* and *FOXJ1* correlated positively with the pattern scores of *RSPH1*, *RSPH4A*, *RSPH4A,* and *DNAH5* (all *P* < 0.05, **Figure 4**). Similarly, the mRNA levels of *CP110* also correlated positively with those of ultrastructural markers (all *P* < 0.001). *FOXJ1* mRNA expression also correlated positively with that of *RSPH1* and *DNAH5* (*r* = 0.706, *P* < 0.001; *r* = 0.213, *P* = 0.044), but not *RSPH4A* and *RSPH9* (*r* = 0.131, *P* = 0.218; *r* = 0.046, *P* = 0.667) (**Figure S2**). In the NP-only groups, the TFI of *CP110* correlated significantly with the pattern score of *RSPH1, RSPH4A, RSPH9,* and *DNAH5* (all *P* < 0.05), but not the TFI of *FOXJ1* (*P* > 0.05, **Figure S4**).

### Cilia Length of Primary Single Cells and Cilia Ultrastructure Assessment Under TEM

Among single-cell cytospin preparations, the mean of cilia length was significantly greater in NP than in IT (6.5 ± 0.9µm vs. 4.65 ± 0.7µm, *P* < 0.05) (**Figure 4**). Non-significantly greater cilia length was observed in eosinophilic NP compared with non-eosinophilic NP (6.6 ± 1.0µm vs. 6.4 ± 0.9µm, *P* = 0.481). Neutrophilia did not contribute to significantly different cilia length (**Figure S6**).

**Figure 7:**
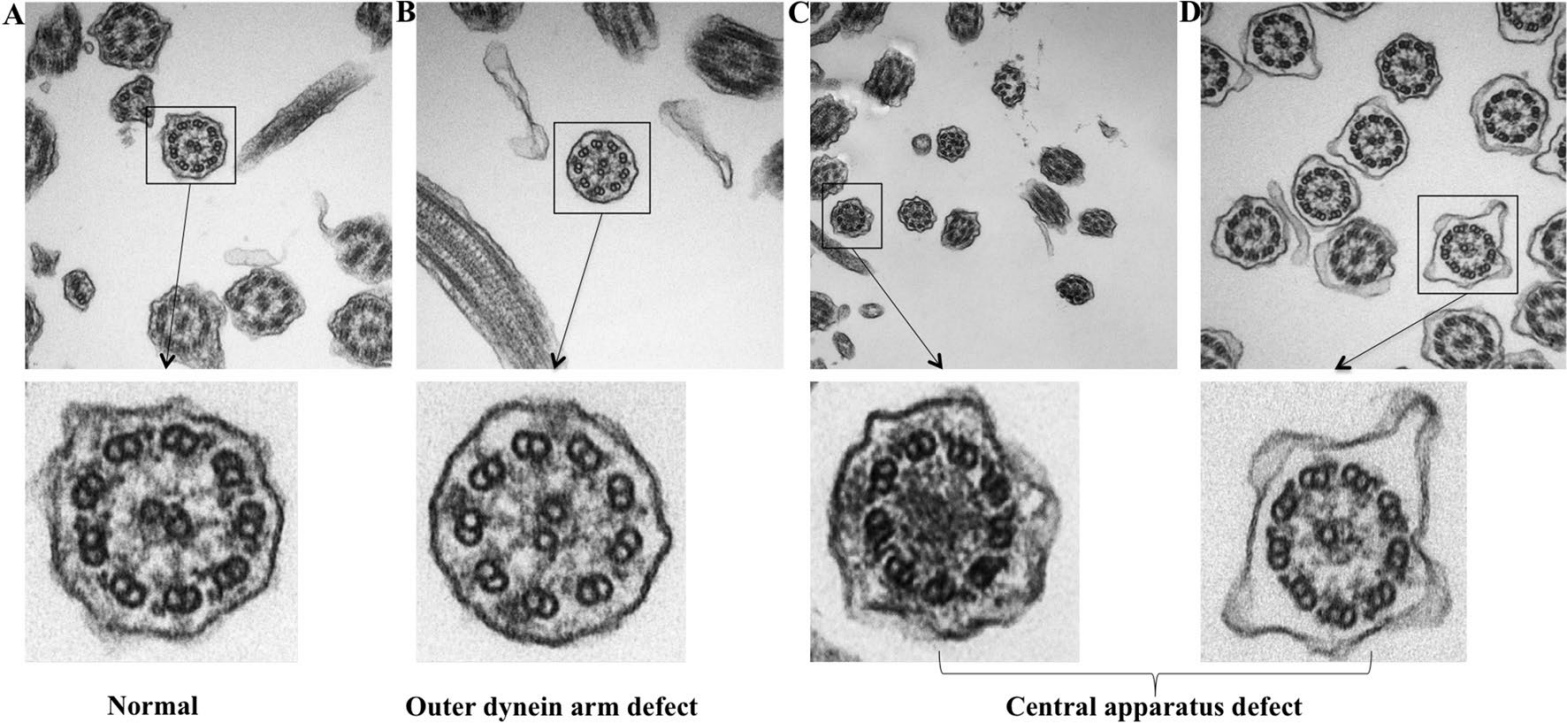
TEM demonstrating the ultrastructural findings in control and NP subject. **(A)** Normal cilia ultrastructure from control subject (original magnification 100,000). **(B)** ODA defect from NP subject (original magnification 100,000). **(C**–**D)** Miss central pair from NP subject (original magnification 50,000 and 100,000). TEM: Transmission electron microscope; ODA, Outer dynein arms.

Because TEM has been applied as the standardized diagnostic tool for ciliary disorders in clinical settings, we have further evaluated the IT and NP samples with TEM to provide objective evidence that the insults of airway inflammation could have predisposed the epithelium to secondary ciliary disorders with an IF staining-independent technique. Indeed, under TEM, normal cilia ultrastructure was found in control epithelium, while abnormal cilia ultrastructure was observed in NP samples, including the outer dynein arm defects and central pair defects (**Figure 7**). These findings reaffirmed that the ultrastructural defects which are verified by TEM can be detected with immunofluorescence staining.

### Increased Abnormal Expressions of Ciliary Ultrastructural and Ciliogenesis Markers in Eosinophilic NP

**Figure 5:**
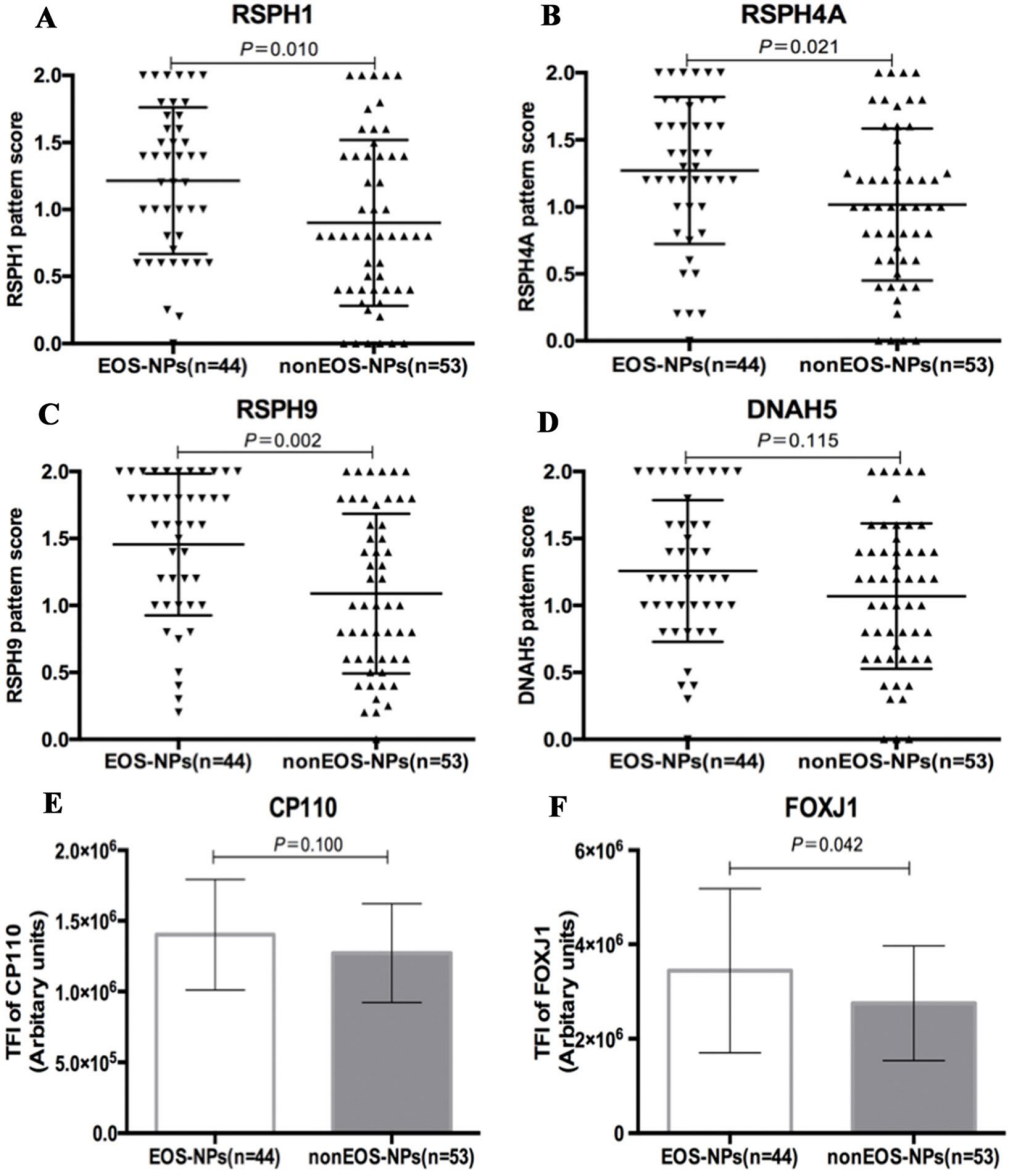
Comparison of the expression levels of *RSPH1*, *RSPH4A*, *RSPH9*, *DNAH5*, *CP110,* and *FOXJ1* based on the airway inflammatory phenotypes. **(A–D)** The different expression pattern scores of ciliary ultrastructure markers (*RSPH1*, *RSPH4A*, *RSPH9,* and *DNAH5*) in eosinophilic and non-eosinophilic nasal polyps. **(E–F)** The different expression levels of ciliogenesis markers in eosinophilic and non-eosinophilic nasal polyps. *RSPH*, Radial spoke head protein; *DNAH5*, Dynein arm heavy chain 5; *CP110*, Centrosomal protein 110; *FOXJ*, Fork-head box protein J1, DAPI, 4’,6-diamidino-2-phenylindole. The values are expressed as Mean and standard deviation.

We further stratified NP into eosinophilic and non-eosinophilic NP. In eosinophilic NP, the median pattern scores of *RSPH1*, *RSPH4A,* and *RSPH9* were significant higher than non-eosinophilic NP [1.3 (0.7, 1.7) vs. 0.8 (0.4, 1.4), 1.3 (1.0, 1.7) vs. 1.0 (0.6, 1.4), and 1.6 (1.0, 2.0) vs. 1.0 (0.6, 1.7), all *P* < 0.05]. However, the pattern score of *DNAH5* was non-significantly higher in eosinophilic NP [1.2 (1.0, 1.6) vs. 1.2 (0.6, 1.4), *P* = 0.115] (**Figure 5**). In light of the positive relationships among these ultrastructural markers and the limited single-cell slide samples, we only further analyzed the subgroups of *DNAH5* staining. The percentage of pattern A, B, and C of *DNAH5* single-cell staining was 43.3%, 15.6%, and 41.1% in eosinophilic NP. Correspondingly, the figures in non-eosinophilic NP were 58.2%, 11.8%, and 30.0% (*P* = 0.112), respectively (**Table S2**).

Compared with non-eosinophilic NP, eosinophilic NP yielded non-significantly higher TFI of *CP110* [1.4 (1.2, 1.6) vs. 1.2 (1.0, 1.5) ×10^6^ arbitrary units, *P* = 0.100]. The median TIF of *FOXJ1* was significantly higher in eosinophilic NP [3.1 (2.2, 4.6) vs. 2.6 (2.1, 3.0) ×10^6^ arbitrary units, *P* < 0.01] (**Figure 5**).

## Discussion

The human nasal epithelium constitutes the first-line defense against pathogens and has self-repairing capabilities that are critical for maintaining homeostasis of mucosal microenvironment (Duan et al., 2016). However, in NP there may be the interplay between the chronic airway inflammation and motile ciliary disorders (Al-Rawi et al., 1998; Gudis et al., 2012). Our study has reaffirmed the intimate association between defective ciliogenesis and abnormal expression of ciliary ultrastructural marker, and highlighted the roles of chronic inflammation in driving abnormal ciliary ultrastructural marker expression and defective ciliogenesis in NP that is not associated with congenital disorders such as PCD. Eosinophilic inflammation might have a role in dampening mucociliary clearance.

In healthy subjects, 4–10% of respiratory cilia reportedly demonstrated ultrastructural abnormalities (Smallman and Gregory 1986; de Iongh and Rutland, 1995; Bush et al., 1998). Nonetheless, abnormal cilia can be found in ∼20% of patients with chronic inflammatory airway diseases such as chronic rhinosinusitis (CRS) (Al-Rawi et al., 1998; Plesec et al., 2008). Intriguingly, 87% of patients with severe CRS reportedly had compound cilia and microtubule and dynein arm defects (Al-Rawi et al., 1998). Truncation or absence of inner or outer dynein arms were the most common pattern (∼71%) in respiratory cilia with abnormal ciliary ultrastructure, and 19% of these patients may have exhibited RS defects (only one patient reportedly had PCD) (Plesec et al., 2008). Consequently, outer dynein arm and RS protein markers might be useful for assessment of ciliary ultrastructural abnormalities in chronic inflammatory airway diseases. In our study, the prevalence of abnormal expression of all ciliary ultrastructural markers (*RSPH1*, *RSPH4A*, *RSPH9,* and *DNAH5*) was markedly higher and highly correlated. The significant correlation between ultrastructural markers suggested that ultrastructural marker assemblies might be affected to a similar magnitude. Therefore, cilia length was significantly greater in NP than in IT (particularly in eosinophilic NP), suggesting that prolongation of the axoneme and assembly of ultrastructural markers was simultaneously affected, thus contributing to abnormal ciliary function. These findings have expanded our understanding that absence and/or mislocalization of ciliary ultrastructural markers is common in NP (Qiu et al., 2018). Interestingly, despite the greater prevalence of absence of axonemal proteins, the mRNA expression levels of most ultrastructural markers we investigated in this study (*RSPH4A, RSPH9* and *DNAH5*) were significantly higher in NPs. Consistent with the current findings, in light of the longer and denser cilia in NP, we hypothesized that the abnormal up-regulated ciliogenesis (possibly because of chronic airway inflammation) might have contributed to the abnormal expression patterns of ultrastructural markers and that these might be associated with eosinophilic inflammation.

*CP110* is localized to the cilia-forming basal bodies and is indispensable to the formation and proper functioning of respiratory cilia. Thus, *CP110* may have contributed to the modulation of cilia length (Walentek et al., 2016). Inflammation-mediated up-regulation of CP110 expression, which correlated with poor ciliogenesis, was reportedly common in NP (Lai et al., 2011; Li et al., 2014). Consistently, our study revealed that the protein and mRNA expression levels of *CP110* in NPs were increased, which was more prominent in eosinophilic NP. The increased *CP110* expression correlated with the abnormal expression patterns of all ultrastructural markers and *FOXJ1*. Greater cilia length reportedly correlated with the up-regulated *CP110* levels in both upper and lower airway diseases (Li et al., 2014; Chen et al., 2018). Therefore, the abnormal expression of ciliogenesis markers (i.e. *CP110*) might drive motile ciliary disorders in chronic airway inflammatory diseases due to abnormal cilia length that affects assembly of ciliary ultrastructural proteins.

*FOXJ1* is highly expressed in ciliated cells and is prerequisite for cilia formation (Blatt et al., 1999; You et al., 2004). Decreased *FOXJ1* expression correlated with loss of respiratory cilia, whereas up-regulated *FOXJ1* expression correlated with ultrastructural marker abnormality (Look et al., 2001). *FOXJ1* reportedly activated the gene expression encoding motile ciliary markers, including heavy chain subunits and RS proteins (Stubbs et al., 2008). Additionally, increased expression of *FOXJ1* in NP resulted in lengthened or overly dense cilia, leading to impaired ciliary motility (Li et al., 2014; Jiao et al., 2016). In our study, the heightened *FOXJ1* expression levels correlated with a greater prevalence of absence of ciliary ultrastructural markers in Nasal specimens. In contrast to previous reports (Li et al., 2014; Jiao et al., 2016), *FOXJ1* mRNA levels were non-significantly greater and the correlation between *FOXJ1* and ciliary ultrastructure markers were non-significant in the NP-only group. Nonetheless, the aberrant localization rather than the non-significant increase of *FOXJ1* would be of greater clinical relevance to the pathogenesis of NPs. Recently, we have identified four distinct *FOXJ1* localization patterns in allergic nasal mucosa that partially correlated with allergic airway inflammation (Peng et al., 2018). Further investigation exploring how mislocalization of *FOXJ1* contributes to the abnormal expression patterns of ciliary ultrastructural markers is warranted.

**Figure 6:**
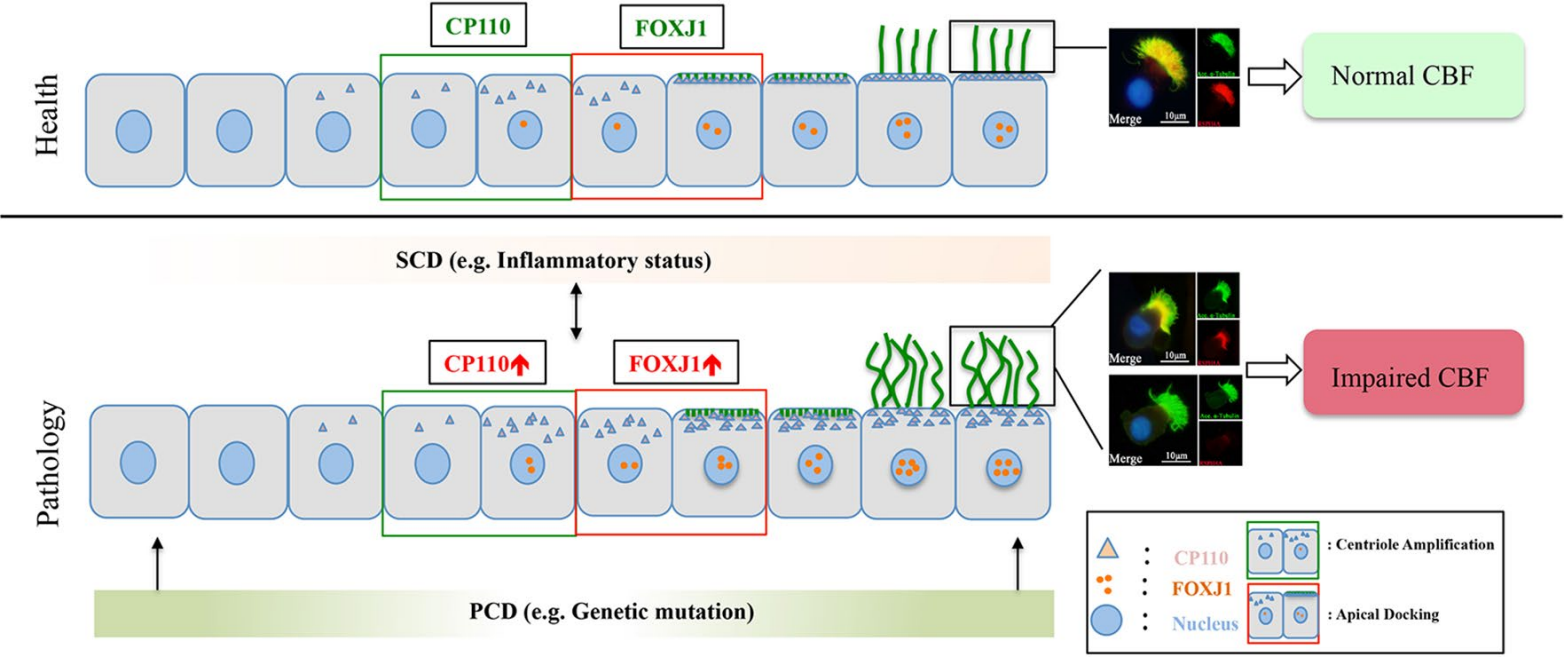
A schematic diagram demonstrating the roles of chronic airway inflammation in driving aberrant ciliogenesis and ultrastructural markers. In health, the differentiation of ciliated cells is under delicate regulation of *CP110* and *FOXJ1* which are responsible for normal assembly of ultrastructural markers. Upon chronic inflammation, up-regulated *CP110* and *FOXJ1* expression contributes to the overly dense and lengthened cilia, which correlates with abnormal expressions and localization of ciliary ultrastructural markers, leading to SCD. Conversely, SCD may disrupt ciliogenesis and result in abnormal expression of ciliary ultrastructural markers (i.e., *RSPH4A*). Contrarily, PCD mainly stems from genetic defects that disrupt cilia assembly at early stages of cell differentiation. For the purpose of illustrating the expression patterns of ciliary markers (i.e., *FOXJ1*), we have only demonstrated the ciliated cells in this schematic diagram. PCD, primary ciliary dyskinesia; SCD, secondary ciliary dyskinesia.

Collectively, in the milieu of the chronic inflammation, both abnormal ciliogenesis and ciliary ultrastructure (i.e., *DNAH5* defects) are responsible for the manifestation of impaired MCC in NP (particularly eosinophilic NP). Upon stimulation of the chronic inflammation, up-regulated *CP110,* and *FOXJ1* expression may contribute to the overly dense and increased cilia length, and the abnormal expression and localization of ciliary ultrastructural markers, resulting in disrupted cilia assembly and affecting ciliary motility (**Figure 6**).

Although in the present study the ciliary ultrastructural defects could be detected with TEM (**Figure 7**), it should be stressed that TEM is not the ‘gold standard’ for the diagnosis of ciliary disorders, which may be particularly true when the outer or inner dynein arm defects could not be displayed clearly. Some ultrastructural protein mutations might have resulted in a completely normal ciliary ultrastructure. For instance, mutations of RSPH9 might have resulted in completely normal ciliary ultrastructure, RSPH4A mutations might have resulted in approximately 50% of the cilia with normal ultrastructure, and RSPH1 mutation might have led to an overall normal TEM image. Because TEM may be laborious and technically challenging as a diagnostic tool, we have now resorted to immunofluorescence staining that has been increasingly validated as the practical surrogate for assessment of ciliary disorders for the diagnosis.

The study is not without limitations. First, we did not sample nasal mucosa of the middle turbinate or ethmoid mucosa from control subjects for comparison. Ideally, sampling the tissues from the uncinate process as the control would be preferred. Nonetheless, this approach for the healthy controls appeared to be unethical, according to the feedback from our ethics review board. In fact, the turbinate tissue has already been commonly used as the control tissue to represent healthy controls in assessing tissue histology in literature reports, which partially validated our sampling approaches. Contrarily, sampling IT tissues is practical and significantly less invasive. The samples from the nasal cavity are lined with, or covered by, the same type of pseudostratified columnar respiratory epithelium, including ciliated cells, non-ciliated cells, goblet cells, and basal cells. Additionally, we also showed that IT tissues provides comparable readouts as the middle turbinate and uncinate process in cilia architecture in one of our previous publication; thus rendering it particularly suitable for translational cilia research (Li et al., 2014; Duan et al., 2016; Zhao et al., 2017; Qiu et al., 2018). Second, the cilia length in paraffin-embedded tissues was not measured. However, the bending or distortion during sample processing could have confounded our measurement. Third, we did not provide a more direct evidence to confirm the relationships between the chronic airway inflammation and ciliary ultrastructural abnormality and genetic (primary) defects, which warranted further investigations. Fourth, we noted a discordant trend of changes in IF and qPCR finding. Nonetheless, the significantly greater ciliary length and ciliated area in NP (Li et al., 2014) might be responsible for the increase in mRNA expression levels of ciliary markers. Despite the development of semi-quantitative scoring system that helped evaluate the correlation between ultrastructural defects and ciliogenesis, the current classification scheme remains arbitrary. Further investigation of the clinical relevance is warranted. Fifth, multicenter studies with larger sample sizes are needed. Moreover, we did not employ enzyme-linked immunosorbent assay or western blotting for analysis of protein expression. However, immunofluorescence imaging may not only provide relative quantitative measure (i.e. the TFI) but can also reveal mislocalization of ciliogenesis or partial absence of ciliary ultrastructural markers. Finally, *in vitro* investigation is merited to determine the mechanisms underlying the correlation between expression levels of ultrastructural markers and ciliogenesis markers. Overall, the present study has extended our previous publications in that we have now focused on the defective expression patterns of individual cilia ultrastructural markers, including outer dynein arms and radial spoke head defects (which have been rarely reported) and mislocalization. Furthermore, we have now offered further evidence that insults of airway inflammation could have predisposed to secondary ciliary disorders that could be assessed with IF staining.

In conclusion, a greater prevalence of absence of *RSPH1*, *RSPH4A*, *RSPH9,* and *DNAH5* expressions is observed in NP, which is associated with the up-regulation of ciliogenesis markers (*CP110* and *FOXJ1*) and greater cilia length in the chronic inflammatory milieu. Our integrated findings may help elucidate the roles and events leading to the manifestation of impaired ciliogenesis that may lead to cilia ultrastructural abnormalities in driving NP formation. Given the chronic inflammation and significant tendency of recurrence even after surgery, impaired ciliary-mediated MCC should be comprehensively appraised and managed as an important therapeutic strategy to restore ciliary functions for patients with NP.

## Ethics Statement

Our study was carried out in accordance with The Declaration of Helsinki. Ethics approval was obtained from the institutional review boards of the two participating hospitals. All participants signed written informed consent.

## Author Contributions

Conception and design: LS, D-y W, X-x Z, W-j G. Analysis and interpretation: X-x Z, KS T, W-j G, JL, YP. Collection of the samples: X-x Z, YP, JL, Y-k O, MT. Conducting immunofluorescence and immunohistochemistry experiments: X-x Z, W-j G. TEM experiment: T-t H. Drafting the manuscript for important intellectual content: all authors.

## Funding

This study was supported by grants from the National Medical Research Council No. NMRC/CIRG/1458/2016 (to D-YW), The Major Research Development Program of Shandong Province No. 2016GSF201084 (to LS), National Nature Science Foundation of China No. 81670909 and NO.81873692 (to LS), The Key Research Development Program of Shandong Province No. 2018CXGC1214 (to LS), and Pearl River S&T Nova Program of Guangzhou No. 201710010097 and Guangdong Province Universities and Colleges Pearl River Scholar Funded Scheme 2017 (to W-JG). Dr Kai Sen Tan is a recipient of fellowship support from the EAACI Research Fellowship 2019 (to KST).

## Conflict of Interest

The authors declare that the research was conducted in the absence of any commercial or financial relationships that could be construed as a potential conflict of interest.

## Supplementary Material

Figure S1 Expression patterns of RSPH1 and RSPH4A in primary single-cell cytospin slides and paraffin slides. RSPH1and RSPH4A shared three patterns, which were defined as Pattern A, markers located throughout the entire axoneme (A1-A2, D1-D2); Pattern B, markers partly missing at the distal parts of the axoneme (B1-B2, E1-E2). Pattern C, markers completely missing throughout the entire axoneme (C1-C2, F1-F2). RSPH1 and RSPH4A: Red; alpha-tubulin: Green; DAPI: Blue; Co-localization of RSPH1 and RSPH4A with alpha-tubulin: Yellow. RSPH = Radial spoke head protein, DAPI = 4',6-diamidino-2-phenylindole.

Figure S2 Correlation between ciliary ultrastructural and ciliogenesis markers in mRNA level. Correlations between the mRNA expression of RSPH1, RSPH4A, RSPH9 and DNAH5 are shown in panel A and B. The correlation between the expression of ciliagenesis markers (CP110 and FOXJ1) and ciliary ultrastructual markers are demonstrated in panel C and D. RSPH = Radial spoke head protein; DNAH5 = Dynein arm heavy chain 5; CP110 = Centrosomal protein 110; FOXJ = Fork-head box protein J1, DAPI = 4',6-diamidino-2-phenylindole.

Figure S3 The abnormal expressions of ciliary ultrustructural markers and their relationships in nasal specimens. (A, B) Corerlation between the pattern socres of RSPH1, RSPH4A, RSPH9 and DNAH5 in NP. (C, D) Corerlation between the pattern socres of RSPH1, RSPH4A, RSPH9 and DNAH5 in IT. RSPH = Radial spoke head protein; DNAH5= Dynein arm heavy chain 5; CP110 = Centrosomal protein 110; FOXJ = Fork-head box protein J1, DAPI = 4',6-diamidino-2-phenylindole.

Figure S4 The correlation between ciliary ultrastructual markers and ciliogenesis markers in nasal specimens (A, B) Corerlation between the TFI of ciliogenesis markers (CP110 and FOXJ1) and ciliary ultrastructual markers (RSPH1, RSPH4A, RSPH9 and DNAH5) in NP. (C, D) Corerlation between the TFI of ciliogenesis markers (CP110 and FOXJ1) and ciliary ultrastructual markers (RSPH1, RSPH4A, RSPH9 and DNAH5) in IT. RSPH = Radial spoke head protein; DNAH5 = Dynein arm heavy chain 5; CP110 = Centrosomal protein 110; FOXJ = Fork-head box protein J1, DAPI = 4',6-diamidino-2-phenylindole.

Figure S5 Comparison of the expression levels of RSPH1, RSPH4A, RSPH9, DNAH5, CP110 and FOXJ1 based on the neutrophilic status. (A-D) The different expression pattern scores of ciliary ultrastructure markers (RSPH1, RSPH4A, RSPH9 and DNAH5) in neutrophilic and non-neutrophilic NP. (E-F) The different expression levels of ciliogenesis markers in neutrophilic and non-neutrophilic NP. RSPH = Radial spoke head protein; DNAH5= Dynein arm heavy chain 5; CP110 = Centrosomal protein 110; FOXJ = Fork-head box protein J1, DAPI = 4',6-diamidino-2-phenylindole.

Figure S6 Comparison of the single cilia cell length based on the inflammatory phenotypes. (A) Single cilia cell length was compared in patients with eosinophilic and non-eosinophilic NP (B) Single cilia cell length was compared in patients with neutrophilic and non-neutrophilic NP.
