# Supplementary figures and images for: An Integrated Analysis of Radial Spoke Head and Outer Dynein Arm Protein Defects and Ciliogenesis Abnormality in Nasal Polyps
Source: Front Genet. 2019 Nov 13;10:1083. doi: 10.3389/fgene.2019.01083 (PMC6863926; doi:10.3389/fgene.2019.01083)

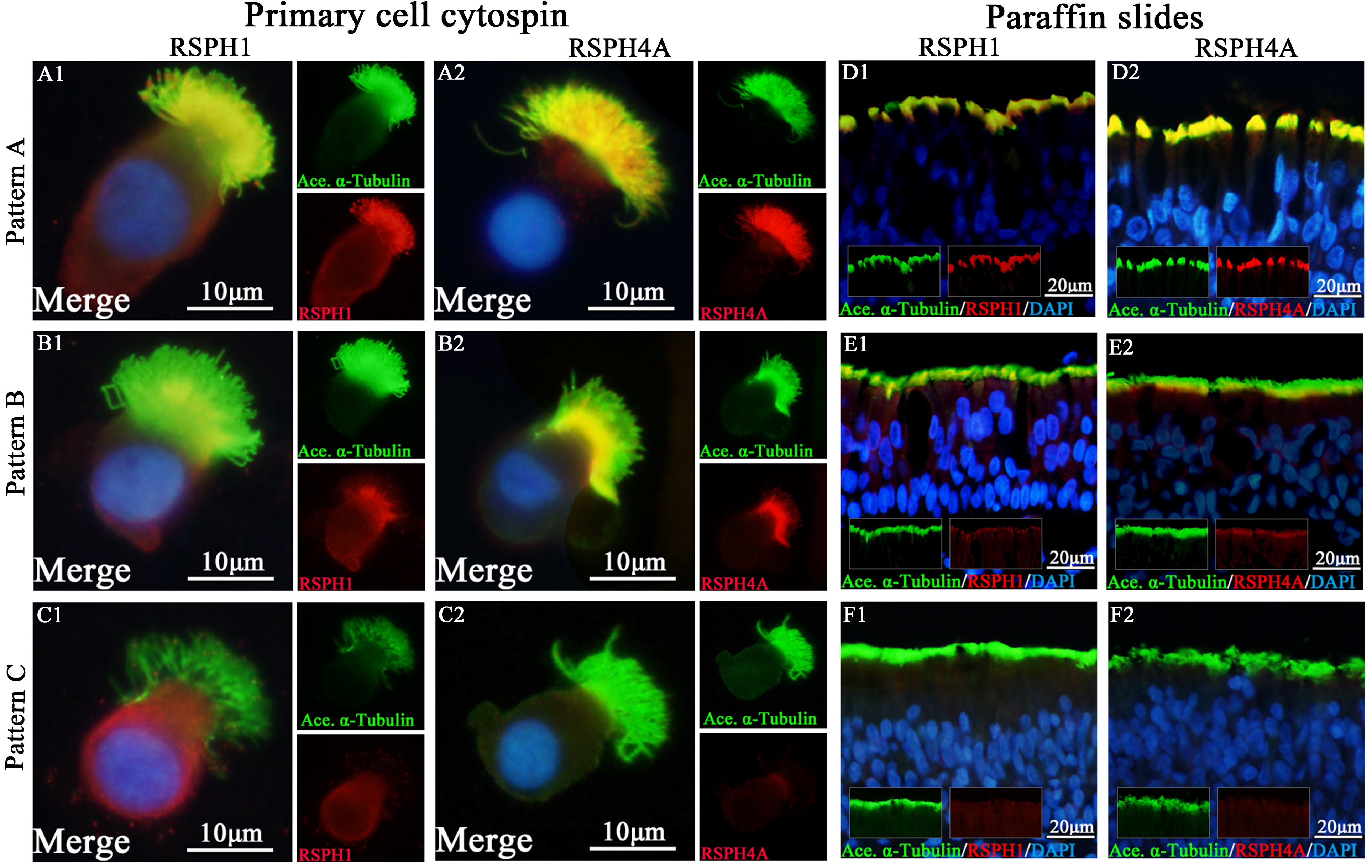

Supplement: Figure S1 — Expression patterns of RSPH1 and RSPH4A in primary single-cell cytospin slides and paraffin slides. RSPH1and RSPH4A shared three patterns, which were defined as Pattern A, markers located throughout the entire axoneme (A1-A2, D1-D2); Pattern B, markers partly missing at the distal parts of the axoneme (B1-B2, E1-E2). Pattern C, markers completely missing throughout the entire axoneme (C1-C2, F1-F2). RSPH1 and RSPH4A: Red; alpha-tubulin: Green; DAPI: Blue; Co-localization of RSPH1 and RSPH4A with alpha-tubulin: Yellow. RSPH = Radial spoke head protein, DAPI = 4',6-diamidino-2-phenylindole. [file Image_1.jpg]

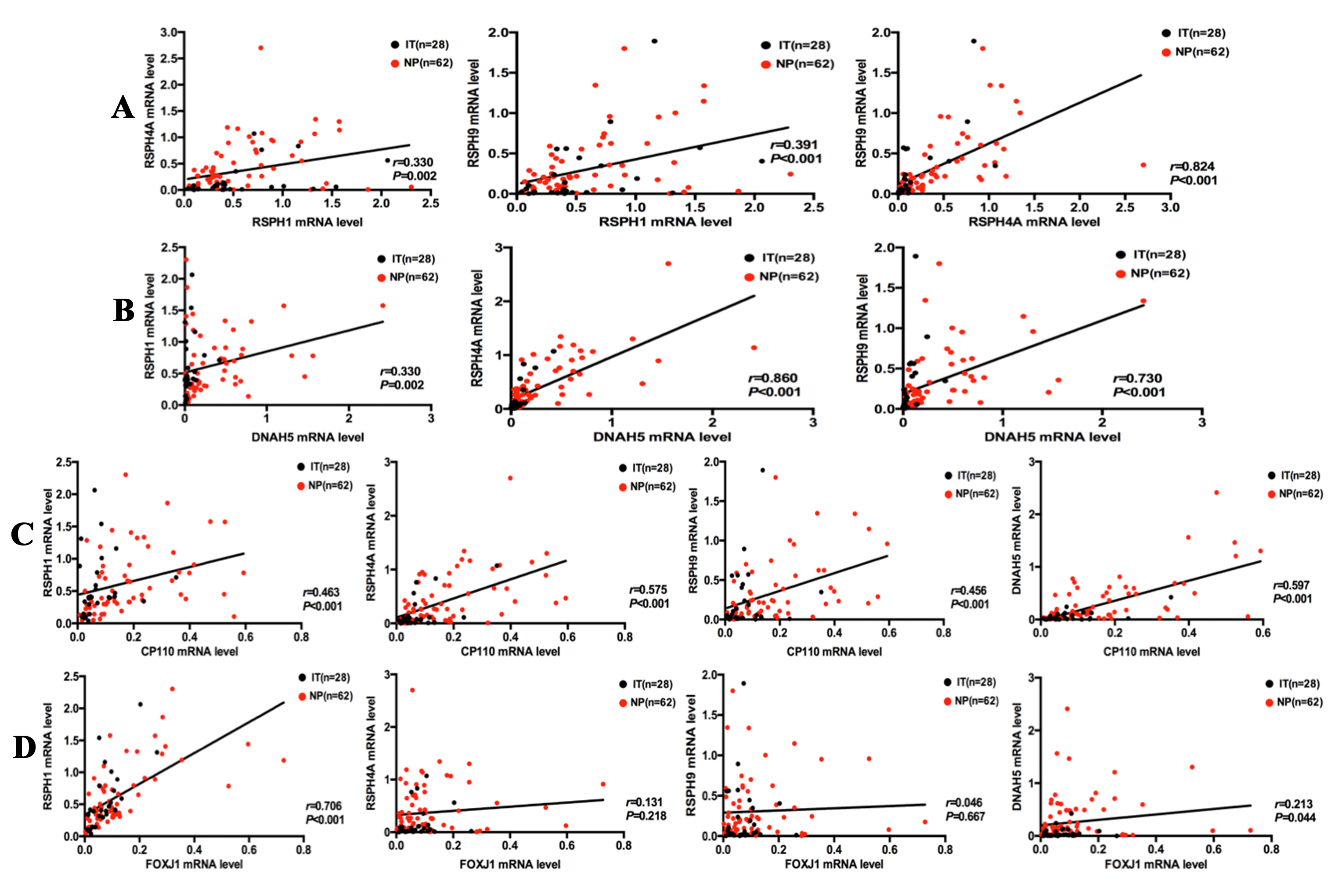

Supplement: Figure S2 — Correlation between ciliary ultrastructural and ciliogenesis markers in mRNA level. Correlations between the mRNA expression of RSPH1, RSPH4A, RSPH9 and DNAH5 are shown in panel A and B. The correlation between the expression of ciliagenesis markers (CP110 and FOXJ1) and ciliary ultrastructual markers are demonstrated in panel C and D. RSPH = Radial spoke head protein; DNAH5 = Dynein arm heavy chain 5; CP110 = Centrosomal protein 110; FOXJ = Fork-head box protein J1, DAPI = 4',6-diamidino-2-phenylindole. [file Image_2.jpg]

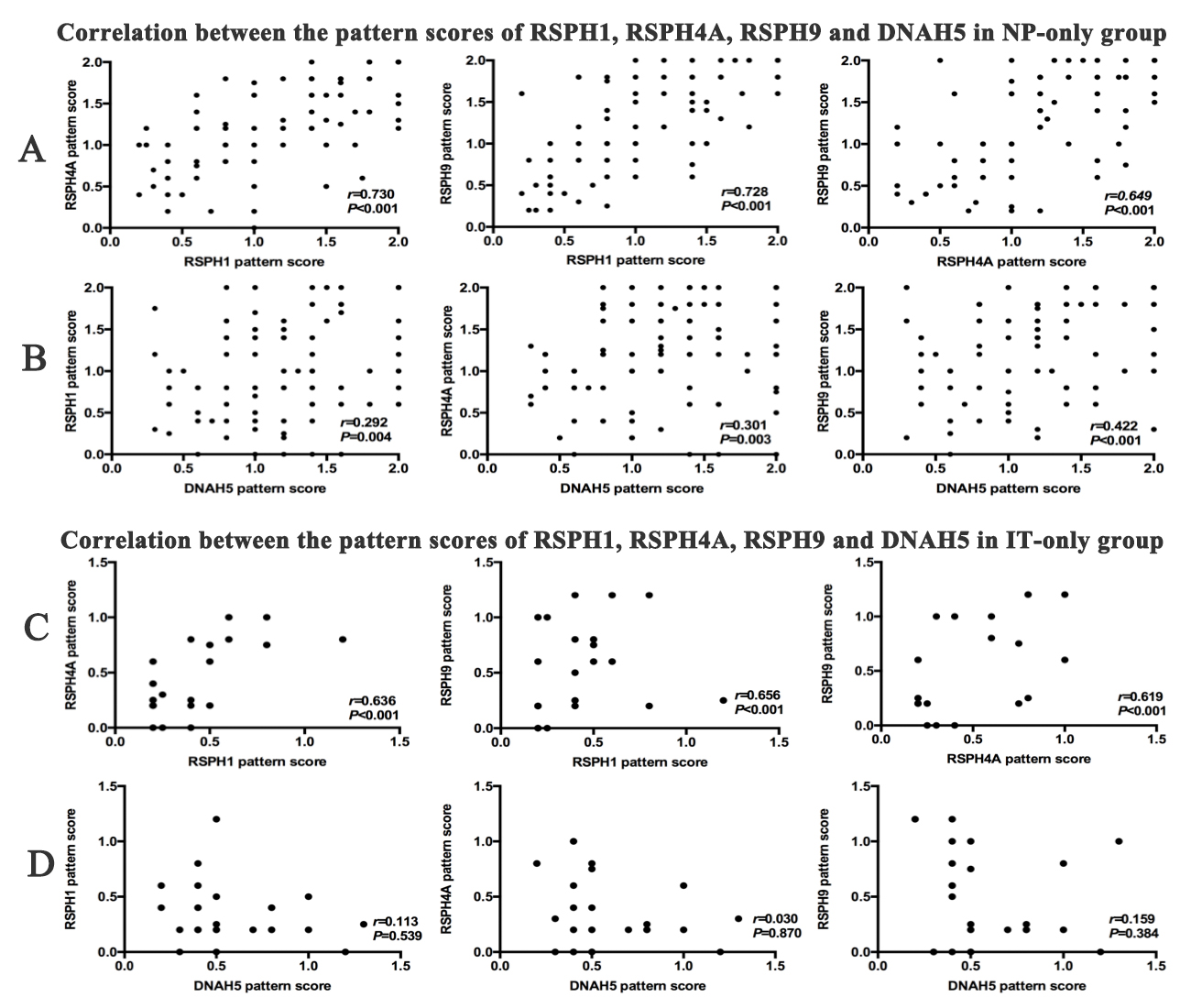

Supplement: Figure S3 — The abnormal expressions of ciliary ultrustructural markers and their relationships in nasal specimens. (A, B) Corerlation between the pattern socres of RSPH1, RSPH4A, RSPH9 and DNAH5 in NP. (C, D) Corerlation between the pattern socres of RSPH1, RSPH4A, RSPH9 and DNAH5 in IT. RSPH = Radial spoke head protein; DNAH5= Dynein arm heavy chain 5; CP110 = Centrosomal protein 110; FOXJ = Fork-head box protein J1, DAPI = 4',6-diamidino-2-phenylindole. [file Image_3.jpg]

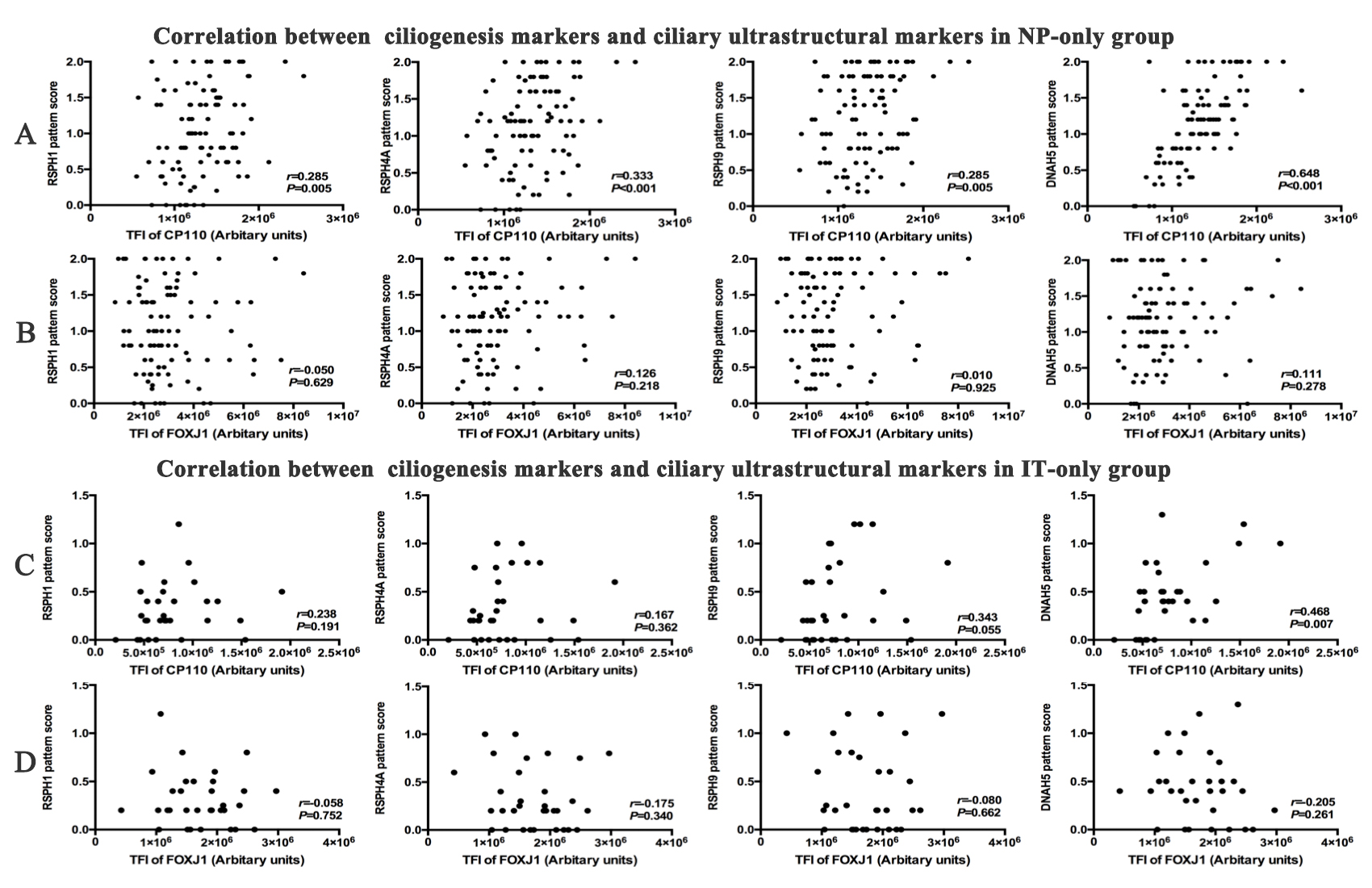

Supplement: Figure S4 — The correlation between ciliary ultrastructual markers and ciliogenesis markers in nasal specimens (A, B) Corerlation between the TFI of ciliogenesis markers (CP110 and FOXJ1) and ciliary ultrastructual markers (RSPH1, RSPH4A, RSPH9 and DNAH5) in NP. (C, D) Corerlation between the TFI of ciliogenesis markers (CP110 and FOXJ1) and ciliary ultrastructual markers (RSPH1, RSPH4A, RSPH9 and DNAH5) in IT. RSPH = Radial spoke head protein; DNAH5 = Dynein arm heavy chain 5; CP110 = Centrosomal protein 110; FOXJ = Fork-head box protein J1, DAPI = 4',6-diamidino-2-phenylindole. [file Image_4.jpg]

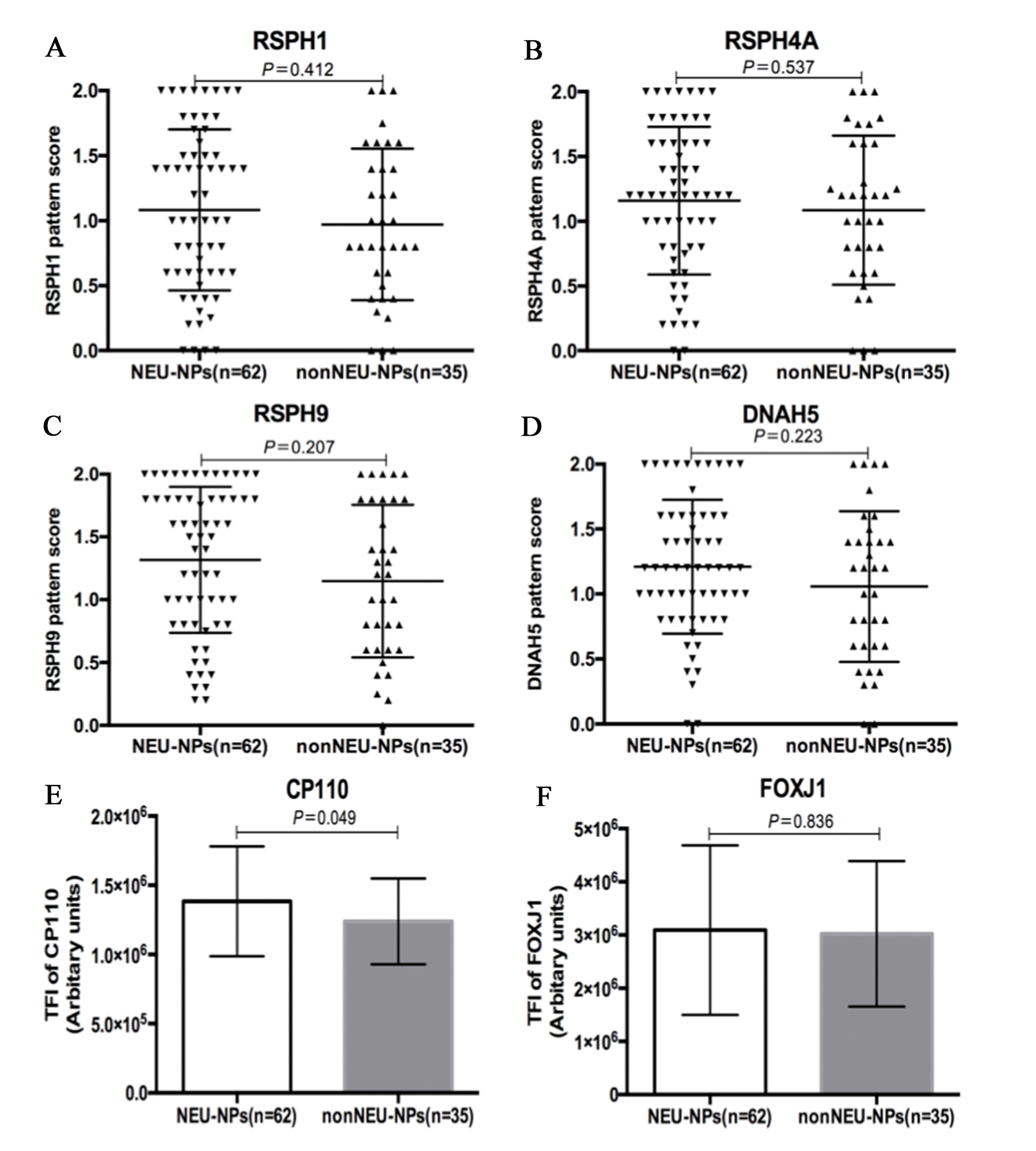

Supplement: Figure S5 — Comparison of the expression levels of RSPH1, RSPH4A, RSPH9, DNAH5, CP110 and FOXJ1 based on the neutrophilic status. (A-D) The different expression pattern scores of ciliary ultrastructure markers (RSPH1, RSPH4A, RSPH9 and DNAH5) in neutrophilic and non-neutrophilic NP. (E-F) The different expression levels of ciliogenesis markers in neutrophilic and non-neutrophilic NP. RSPH = Radial spoke head protein; DNAH5= Dynein arm heavy chain 5; CP110 = Centrosomal protein 110; FOXJ = Fork-head box protein J1, DAPI = 4',6-diamidino-2-phenylindole. [file Image_5.jpg]

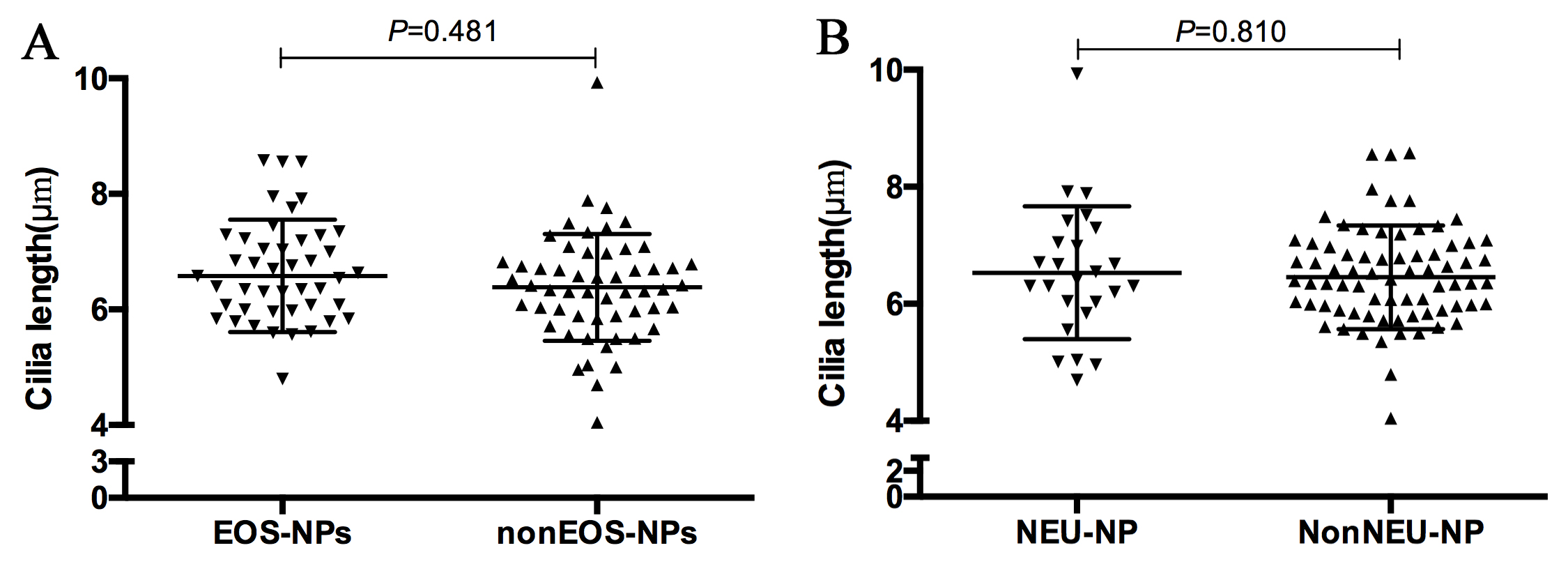

Supplement: Figure S6 — Comparison of the single cilia cell length based on the inflammatory phenotypes. (A) Single cilia cell length was compared in patients with eosinophilic and non-eosinophilic NP (B) Single cilia cell length was compared in patients with neutrophilic and non-neutrophilic NP. [file Image_6.jpg]
