# Supplementary material for: An Integrated Analysis of Radial Spoke Head and Outer Dynein Arm Protein Defects and Ciliogenesis Abnormality in Nasal Polyps
Source: Front Genet. 2019 Nov 13;10:1083. doi: 10.3389/fgene.2019.01083 (PMC6863926; doi:10.3389/fgene.2019.01083)
Supplement: Supplementary file 7 [file DataSheet_1.pdf]

Online Repository

An integrated analysis of radial spoke head and outer dynein arm protein defects and ciliogenesis abnormality in nasal polyps

Xiao-xue Zi[1,2*] , Ph.D.; Wei-jie Guan[2,3*], Ph.D.; Yang Peng[2,3] , Ph.D; Kai Sen Tan[2] , Ph.D.; Jing Liu[2], Ph.D.; Ting-ting He[2], M.D.; Yew-kwang Ong[4], M.D.; Mark Thong[4], M.D.; Li Shi[1] , M.D., Ph.D.; De-Yun Wang[2] , M.D., Ph.D.

[1] Department of Otolaryngology-Head and Neck Surgery, Shandong Provincial ENT Hospital Affiliated to Shandong University, Jinan 250022, P.R. China

[2] Department of Otolaryngology, Yong Loo Lin School of Medicine, National University of Singapore, Singapore, Singapore

[3] State Key Laboratory of Respiratory Disease, National Clinical Research Center for Respiratory Disease, Guangzhou Institute of Respiratory Health, First Affiliated Hospital of Guangzhou Medical University, Guangzhou Medical University, Guangzhou, Guangdong, China

[4] Department of Otolaryngology-Head and Neck Surgery, National University Hospital System (NUHS), Singapore, Singapore

[*]: Drs. Xiao-xue Zi and Wei-jie Guan contributed equally.

Corresponding author: Li Shi, M. D., Ph.D., Shandong Provincial ENT Hospital affiliated to Shandong University, Address: 4 Duanxing west road, Jinan, Shandong, China, Fax: +86 531 88962544, Phone: +86 531 85875317, E-mail: shili126@sina.com

Corresponding author: De-Yun Wang, M. D., Ph.D., Department of Otolaryngology, Yong Loo Lin School of Medicine, National University of Singapore, 1E Kent Ridge Rd, Singapore 119228. E-mail: entwdy@nus.edu.sg

Abbreviations: NP = Nasal polyp, IT = Inferior turbinate, RSPH = Radial spoke head protein, DNAH = Dynein arm heavy chain, CP110 = Centrosomal protein 110, FOXJ1 = Fork-head box protein J1, CBF = ciliary beat frequency, MCC = Impaired mucociliary clearance, PCD=primary ciliary dyskinesia, CRSwNP = chronic rhinosinusitis with nasal poly, IHC = Immunohistochemistry, IF = Immunofluorescence, TFI = total fluorescence intensity, MFI = mean fluorescence intensity, qRT-PCR = Quantitative real-time polymerase chain reaction, SCD = secondary ciliary dyskinesias.

Methods

Immunohistochemistry (IHC) assay

Fresh NP and IT biopsy samples were immediately embedded in paraffin. The 4-µm serial sections were obtained from each paraffin blocks with a Leica microtome (Leica, Wetzlar, Germany) for haematoxylin-eosin (H&E) and IHC staining. IHC staining was performed by using the modified horseradish peroxidase technique and the Dako Cytomation EnVision + System (Dako Inc., Denmark). Endogenous peroxidase was blocked with 3% H_2O_2. Slides were incubated with primary antibodies at 4 °C overnight. The slides were incubated with Dako EnVision+System-HRP (Dako) at room temperature for 30 minutes (1-4). Diaminobenzidine was used as a substrate for color development. All slides were counterstained with hematoxylin.

Immunofluorescence (IF) assay

Paraffin tissue sections and cytospin samples were incubated with primary antibodies at 4°C overnight, followed by incubation with Alexa Fluor 488 or 594 conjugated secondary antibodies (Life Technologies, Carlsbad, CA, USA) at 1:500 in the dark for 1 hour at room temperature. We mounted the slides by using SlowFade Gold anti-fade reagent with 4’6-diamidino-2-phenylindole (DAPI; Life Technologies Inc., USA). Double staining was performed in immunofluorescence staining by using antibodies of two different host species. Mouse anti-human alpha-tubulin (1:800, ab24610, Abcam) combined with rabbit anti-human RSPH1, RSPH4A, RSPH9, DNAH5, CP110 and FOXJ1 (primary antibodies)(1-4). Species- and subtype-matched primary antibodies were applied as negative controls. The slides were analyzed with fluorescent microscopy (Olympus IX51, Tokyo, Japan).

RNA extraction and quantitative real-time polymerase chain reaction (qRT-PCR)

Total RNA was extracted from NP and IT tissues in RNAlater (Ambion, Austin, TX, USA) with the mirVana™ isolation kit, followed by reverse transcription to yield complementary DNA (cDNA) by using Maxima First Strand cDNA Synthesis Kits (Applied Biosystems Inc., USA) according to manufacturer’s protocol. The expression levels of ciliary markers primers listed in **Table S1**. Relative gene expression was calculated using the $2^{-\Delta\Delta Ct}$ algorithm with glyceraldehyde-3-phosphate dehydrogenase (GAPDH) as the reference.

Transmission electron microscopy (TEM)

Tissue samples from NP and control subjects were washed twice in PBS, fixed in 2.5% glutaraldehyde and 0.1% Tannic acid in phosphate buffer at 4 ℃ for overnight incubation. Samples were post-fixed in 1% osmium tetroxide and potassium ferrocyanide for 1 hr followed by progressive dehydration with gradually increasing ethanol concentrations (25%, 50%, 75%, 95% and 100%). Samples were then infiltrated with 1:1 (acetone: resin) for 30 min and 1:6 (acetone: resin) for overnight incubation. The samples were finally embedded in fresh resin and harden at 60% for 24 hrs. We made an ultrathin section (70-90 nm) through the central part of the specimens, which were visualized in a transmission electron microscope (JEOL. Model: JEM 1010) at high-power field magnification.

Representative photomicrographs were taken at various angles to effectively display the specimens so that error of assessment which was associated with the tilt of the specimen or other processing artifacts would be minimized.

Results

Association between ciliary ultrastructural and ciliogenesis markers

Quantitative PCR

The mRNA expressions of ciliary ultrastructual markers (*RSPH1*, *RSPH4A*, *RSPH9* and *DNAH5*) correlated significantly with each other (all *P*<0.05) (**Figure S2**). Next, we further investigated their correlation with ciliogenesis markers -- *CP110* and *FOXJ1*. As showed in **Figure S2**, the mRNA levels of *CP110* correlated positively with that of *RSPH1, RSPH4A, RSPH9* and *DNAH5*, while *FOXJ1* only correlated significantly with that of *RSPH1* and *DNAH5* (r=0.706, *P*<0.05; r=0.213, *P*<0.05).

Protein expression in NPs and ITs

According to our semi-quantitative scoring system, the expression pattern scores of ciliary ultrastructural markers (*RSPH1*, *RSPH4A*, *RSPH9* and *DNAH5*) correlated positively with each other in NP tissues (all *P*<0.05) (**Figure S3**). However, in IT tissues, these correlations lost statistical significance for *DNAH5*, *RSPH1*, and *RSPH9* (*P*>0.05). (**Figure S3**)

In NP tissues, the TFI of *CP110* positively correlated with the pattern scores of *RSPH1*, *RSPH4A*, *RSPH4A* and *DNAH5* (all *P*<0.05, **Figure S4**). However, in IT tissues, only the pattern score of *DNAH5* correlated significantly with that of *CP110*. However, the TFI of *FOXJ1* showed no significant correlation in both NP and IT biopsy tissues.

Subgroup analysis: eosinophilic vs. non-eosinophilic NP and neutrophilic vs. non-neutrophilic NP

The expression patterns of ciliary ultractructual markers (*RSPH1, RSPH4A, RSPH9* and *DNAH5*) and the TFI of *FOXJ1* were not significantly different between patients with neutrophilic versus nonneutrophilic group (all *P* >0.05), while the TFI of *CP110* in neutrophilic NP was borderline significantly higher than those with neutrophilic NPs (*P*=0.049). (**Figure S5**)

In primary single cilia cells, the percentage of pattern A, B, and C was 43.3%, 15.6%, and 41.1% among patients with eosinophilic NPs, whereas the percentage was 58.2%, 11.8%, and 30.0% of cells among patients with non-eosinophilic NP. Correspondingly, the percentage of pattern A, B, and C was 54.0%, 14.0% and 32.0% from samples with neutrophilic NPs, while the percentage was 50.7%, 13.3% and 36.0% from samples with non-neutrophilic NPs. (**Table S2**)

The cilia length measured in single-cell cytospin did not differ significantly between paitents with eosinophilic NP and non-eosinophilic NP, as did in neutrophilic when compared with non-neutrophilic subgroups (all P>0.05). (**Figure S6**)

Reference

1. Yan Y, Tan KS, Li C, et al. Human nasal epithelial cells derived from multiple subjects exhibit differential responses to H3N2 influenza virus infection in vitro. J Allergy Clin Immunol 2016;138:276–281.

2. Zhao L, Li YY, Li CW, et al. Increase of poorly proliferated p631 /Ki671 basal cells forming multiple layers in the aberrant remodeled epithelium in nasal polyps. Allergy 2017;72:975–984.

3. Li YY, Li CW, Chao SS, et al. Impairment of cilia architecture and ciliogenesis in hyperplastic nasal epithelium from nasal polyps. J Allergy Clin Immun 2014;134:1282–1292.

4. Qiu Q, Peng Y, Zhu Z, et al. Absence or mislocalization of DNAH5 is a characteristic marker for motile ciliary abnormality in nasal polyps. Laryngoscope. 2018;128:E97-E104.

Table S1. SYBR Green gene expression assays

Primer	Assay
GAPDH	F: 5'- ACAGTTGCCATGTAGACC<br>R: 5'- TTTTTGGTTGAGCACAGG
RSPH1	F: 5'- GGAAAGAGGAGAAGAGGAAG<br>R: 5'- AATTCAGTGATTTGGGTAGC
RSPH4A	F: 5'- TTTGACACCAATCTCTGAAG<br>R: 5'- TTGTGGAATGAGATTTGAGG
RSPH9	F: 5'- GAATATGAACACACTGAGCTG<br>R: 5'- CTTGATCTGGACCACTATTTC
DNAH5	F: 5'- ACTGATGCAACTAATGAAGC<br>R: 5'- AGTGTAGGAATAGCATCCATC
CP110	F: 5'- CTTAGTAGACAAGGAACCCC<br>R: 5'- CCGCTTTCTTTGGATTTTTC
FOXJ1	F: 5'- GTGAAGCCTCCCTACTC<br>R: 5'- AATTCTGCCAGGTGGG

RSPH = Radial spoke head protein; *DNAH5* = Dynein arm heavy chain 5; *CP110* = Centrosomal protein 110; *FOXJ* = Fork-head box protein J1, DAPI = 4',6-diamidino-2-phenylindole; GAPDH = glyceraldehyde-3-phosphate dehydrogenase.

Table S2. The expression patterns of DNAH5 in subgroups of primary cell specimens

	EOS-NP	nonEOS-NP	*P* value	NEU-NP	nonNEU-NP	*P* value
**DNAH5 staining (No.)**	9	11		5	15	
**Single ciliated cells evaluated (No.)**	90	110	0.112	50	150	0.876
Pattern A	39 (43.3%)	64 (58.2%)		27 (54.0%)	76 (50.7%)	
Pattern B	14 (15.6%)	13 (11.8%)		7 (14.0%)	20 (13.3%)	
Pattern C	37 (41.1%)	33 (30.0%)		16 (32.0%)	54 (36.0%)	

#: Primary cells were obtained from 20 patients, these were divided into 9 EOS-NP and 11 nonEOS-NP, 5 NEU-NP and 15 nonNEU-NP. 10 single ciliated cells were randomly selected for assessment from each sample.

DNAH5 = Dynein arm heavy chain 5; EOS-NP = eosinophilic nasal polyps; NEU-NP = neutrophilic nasal polyps.

Table S3. The association among the expression patterns of ciliary ultrastructural markers

	Normal (0<=score<1)	Abnormal (1<=score<=2)	*P* value
**ciliary ultrastructural markers [no. (%)]**			**0.107**
**RSPH1**	75(58.1)	54(41.9)	-
**RSPH4A**	62(48.1)	67(51.9)	-
**RSPH9**	57(44.2)	72(55.8)	-
**DNAH5**	59(45.7)	70(54.3)	-

Comparison was done with the cutoff of 0<=score<1 (normal) and 1=<score<=2 (abnormal). Chi-square test was performed to analyze the correlation between the ciliary ultrastructural markers. No significant difference were observed between the normal/abnormal distribution among the different markers, further confirming that their expression were highly correlated with each other.

RSPH = Radial spoke head protein; DNAH5 = Dynein axonemal heavy chain 5.

Table S4. Correlation among ciliary ultrastructural and ciliogenesis markers with the bootstrapping analysis

Ciliary ultrastructural and ciliogenesis markers	RSPH4A	RSPH9	DNAH5	FOXJ1	CP110
**RSPH1**	0.813 (0.735,0.872)	0.780 (0.695,0.849)	0.450 (0.315,0.579)	0.206 (0.029,0.385)	0.469 (0.317,0.598)
**RSPH4A**	—	0.746 (0.648,0.827)	0.472 (0.321,0.598)	0.336 (0.158,0.493)	0.501 (0.343,0.621)
**RSPH9**	—	—	0.542 (0.405,0.662)	0.253 (0.089,0.419)	0.484 (0.324,0.611)
**DNAH5**	—	—	—	0.311 (0.129,0.478)	0.746 (0.640,0.825)
**FOXJ1**	—	—	—		0.311 (0.138,0.467)

The samples extracted is 1000 randomization

Shown are the correlation coefficient and the 95% confidence intervals

RSPH = Radial spoke head protein; DNAH5 = Dynein axonemal heavy chain 5.
